# Aminophosphinates against *Helicobacter pylori* ureolysis—Biochemical and whole-cell inhibition characteristics

**DOI:** 10.1371/journal.pone.0182437

**Published:** 2017-08-09

**Authors:** Katarzyna Macegoniuk, Ewa Grela, Monika Biernat, Mateusz Psurski, Grażyna Gościniak, Anna Dziełak, Artur Mucha, Joanna Wietrzyk, Łukasz Berlicki, Agnieszka Grabowiecka

**Affiliations:** 1 Wrocław University of Technology, Faculty of Chemistry, Department of Bioorganic Chemistry, Wrocław, Poland; 2 Medical University of Wrocław, Department of Microbiology, Wrocław, Poland; 3 Institute of Immunology and Experimental Therapy, Polish Academy of Sciences, Wrocław, Poland; Monash University, AUSTRALIA

## Abstract

Urease is an important virulence factor from *Helicobacter pylori* that enables bacterial colonization of human gastric mucosa. Specific inhibition of urease activity can be regarded as a promising adjuvant strategy for eradication of this pathogen. A group of organophosphorus inhibitors of urease, namely, aminophosphinic acid and aminophosphonic acid derivatives, were evaluated *in vitro* against *H*. *pylori* urease. The kinetic characteristics of recombinant enzyme activity demonstrated a competitive reversible mode of inhibition with *K*_*i*_ values ranging from 0.294 to 878 μM. *N*-*n*-Hexylaminomethyl-*P*-aminomethylphosphinic acid and *N*-methylaminomethyl-*P*-hydroxymethylphosphinic acid were the most effective inhibitors (*K*_*i*_ = 0.294 μM and 1.032 μM, respectively, compared to *K*_*i*_ = 23 μM for the established urease inhibitor acetohydroxamic acid).

The biological relevance of the inhibitors was verified *in vitro* against a ureolytically active *Escherichia coli* Rosetta host that expressed *H*. *pylori* urease and against a reference strain, *H*. *pylori* J99 (CagA^+^/VacA^+^). The majority of the studied compounds exhibited urease-inhibiting activity in these whole-cell systems. Bis(*N*-methylaminomethyl)phosphinic acid was found to be the most effective inhibitor in the susceptibility profile studies of *H*. *pylori* J99. The cytotoxicity of nine structurally varied inhibitors was evaluated against four normal human cell lines and was found to be negligible.

## Introduction

*Helicobacter pylori* bacilli are recognized as the most common bacterial agent that causes infections in humans. Colonization with the microorganism is the etiologic factor of chronic antral gastritis, which may have severe consequences in terms of gastric ulcers and/or duodenal ulcer development, mucosa-associated lymphoid tissue (MALT) lymphoma, Ménétrier disease and gastric cancer [1]. *H*. *pylori* was the first bacterial species that was proven to cause cancer, and it is now classified as a group I carcinogen by the International Agency for Research on Cancer [2]. *H*. *pylori*-induced apoptosis of gastric mucosa is linked to cag pathogenicity island expression, VacA cytotoxin release, and a variety of detrimental effects of ureolytic activity [3].

The basic pathophysiological mechanism of urease-driven conditions relies on the direct consequences of the catalyzed hydrolysis of urea to carbon dioxide and ammonia. Protonation of ammonia to ammonium ions causes rapid alkalization that devastates surrounding tissue and body fluids. Local pH increases are traditionally considered an essential factor for *H*. *pylori* to colonize physiologically acidic environments. It is now postulated that rapid urea hydrolysis is also a strategy to control urea concentrations at a level that allows pathogen chemotaxis and recognition of the epithelial surface [4]. Alkalization intensifies, in turn, gastrin secretion and HCl production. Ammonia, along with hydrocarbonate ions that are produced from carbon dioxide by a periplasmic anhydrase, is cytotoxic towards stomach lining cells and enhances the damaging effect of acid and pepsin on stomach epithelium. Recently, it was found that *H*. *pylori*-derived ammonia could lead to monochloramine oxidant formation at the inflammatory site, which might be responsible for the activation of latent Epstein-Barr virus infection in the stomach epithelium [5]. In addition to the ureolysis-driven negative effects of urease activity, several other virulence factors that are connected with the synthesis of bacterial urease protein in human organisms have been described. These factors include urease-mediated recognition and binding to cell-surface glycoconjugates during the colonization of gastric mucin. Beyond the gastrointestinal system, *H*. *pylori* urease induces platelet aggregation and contributes to development of cardiovascular disorders. Moreover, experiments performed on the rat paw edema model indicated the pro-inflammatory activity of purified *Helicobacter* urease. It is also suspected that diseases such as sarcoidosis and rheumatoid arthritis are correlated with urease-positive pathogens. The involvement of bacterial ureases in autoimmune disorders is explained by the molecular mimicry mechanism [6].

*Helicobacter* urease is synthetized constitutively in amounts accounting for approximately 10–15% of the total cell protein. Among other ureases, the *Helicobacter pylori* enzyme is unique with respect to its supramolecular ((αβ)_3_)_4_ structure and higher substrate affinity (*K*_*M*_ of 0.3–0.5 mM) [7]. The majority of this enzyme is located in the cytoplasm (it is not present in the periplasm), with some fraction bound externally to the cell surface. Purified *H*. *pylori* urease has a neutral pH optimum. In intact cells, external urease functions at pH values between 5.0 and 8.5, and internal urease remains active at surrounding pH values as low as 2.5 [8]. The cytoplasmic urease enables the maintenance of a metabolic proton motive force across the inner membrane of the cell despite the acidity of the environment. This gradient is possible due to pH-regulated urea uptake via the proton-gated transporter UreI, which in turn activates cytoplasmic urease as a response to stomach acid secretion [9]. Most discussions in the literature focus on the protective function of internal urease for neutrophilic *H*. *pylori*, which allows the organism’s survival in acid and concomitantly prevents excess alkalization via simple and tight control of urea influx. However, a large amount of urease is produced, and a certain level of activity is present in *H*. *pylori* regardless of the surrounding pH. It was shown that a urease-deficient *H*. *pylori* mutant failed to colonize gnotobiotic piglets, although their gastric acid was artificially neutralized [10]. Moreover, urease is synthetized in all *Helicobacter* strains (including non-gastric species). *H*. *pylori* utilizes urea nitrogen for amino acid synthesis [11], and its urease functionally associates with glutamine synthetase [12]. The latter enzyme is so important in *H*. *pylori* that no regulation system for its deactivation exists in the microorganism. Thus, there is growing evidence for a central role of urease in the nitrogen metabolism of *H*. *pylori* [13].

Though intensive research has been conducted on anti-*Helicobacter* vaccines, eradication can only be accomplished with antibiotics at present [14, 15]. Due to specific mutations that confer antibiotic resistance and allow the occurrence of multistrain infections in one patient, prolonged therapy with at least two antibiotics combined with a proton-pump inhibitor is frequently ineffective. Side effects of excessive use of these medications are also of concern.

Extensive research has been conducted to determine alternative solutions against *Helicobacter* infections. As ureolytic activity is mandatory for this pathogen, urease inhibitors are rational candidates for independent drugs or supporting treatment [16]. The search for novel antiureolytic agents is facilitated by resolving the highly conserved structure of bacterial ureases and the role of two nickel(II) ions in the geometry of the active site and catalysis [17].

Several classes of urease inhibitors have been developed since the crystal structures of bacterial ureases were determined including urea derivatives, quinones, polyphenols, phosphoramidates and hydroxamic acids. Their properties and inhibitory efficiencies were summarized in several review articles [18, 19]. Amides of phosphoric acid (i.e., fluorofamide, N-(diaminophosphinyl)-4-fluorobenzamide) represent the group of urease inhibitors with the highest activity, as they are analogs of the tetrahedral transition state of the urease reaction. Unfortunately, their therapeutic utility is strongly limited due to their low hydrolytic stability (the reported half-life of fluorofamide at pH 2 is 5 min) [20]. Hydroxamic acids exhibit metal-complexing properties and thus constitute powerful inactivators of nickel-dependent urease. This class is represented by acetohydroxamate (AHA), which was approved by the FDA in 1983. The major disadvantage to using acetohydroxamate in treatment is the severe side effects including teratogenicity and psychoneurologic and musculo-integumentary symptoms, and concerns have arisen regarding its safety. Benzoimidazoles (i.e., omeprazole, rabeprazole and lansoprazole) were approved by the FDA in 1999. Though their primary therapeutic activity relies on irreversible inhibition of the H^+^/K^+^-ATPase proton pump in stomach lining cells, they also inhibit bacterial urease by covalent modifications of cysteine residues and interactions with sulfhydryl moieties of the enzyme [21]. Their antimicrobial properties are not sufficient to provide monotherapy, but they are considered an indispensable element of successful treatment of *H*. *pylori* infections [22].

Organophosphorus inhibitors of bacterial ureases originated from aminomethyl-*P*-methylphosphinic acid, a molecule whose structure that can be considered an extended analog of the transition state of enzymatic urea hydrolysis. Optimization of the lead structure yielded efficient inhibitors of *Sporosarcina pasteurii* and *Proteus mirabilis* ureases; these inhibitors comprise three classes of compounds, namely, aminomethyl-*P*-methylphosphinates, aminomethyl-*P*-hydroxymethylphosphinates, and bis(aminomethyl)phosphinates [23–27].

The activity of the previously described aminophosphinic and aminophosphonic acids inspired us to undertake further studies regarding the kinetic properties of this group of inhibitors against purified *H*. *pylori* enzyme. In this paper, dissociation constants for twenty-three selected structures are presented. Whole-cell inhibition studies demonstrate the ability of organophosphorus compounds to diffuse into a model gram-negative microorganism (*E*. *coli*) and reduce the ureolytic activity of the reference (CagA^+^, VacA^+^) *H*. *pylori* strain, J99, *in vitro*.

## Materials and methods

### Regulation of urease expression

Urease from *Helicobacter pylori* strain G27 (National Center for Biotechnology Information code NC_011333.1) was produced in the *E*. *coli* strain Rosetta2 (DE3) Singles F2 ompT hsdS_B_(rB 2 mB 2) gal dcm(DE3) pRARE2 (Cam^R^) (Novagen-Merck Biosciences, Poland). The strain was grown in Luria-Bertani (LB) medium purchased from Biocorp (Poland).

Competent *E*. *coli* Rosetta cells were transformed using the plasmid *pGEM*:*ureOP* [28] according to the standard protocols described by Sambrook [29] and spread on LB plates containing 100 μg/mL ampicillin (Polfa, Tarchomin, Poland). The plates were incubated at 37°C overnight. To evaluate protein expression, a single colony was inoculated into 10 mL of fresh LB broth that was supplemented with 100 μg/mL ampicillin, and the culture was grown at 37°C overnight. This starter culture was then used to inoculate 150 mL of the same LB-ampicillin medium, which was then incubated at 37°C in an orbital shaking incubator (180 rpm) until the optical density at 650 nm reached 0.6 (OD_650_ = 0.6). Subsequently, 96 different induction conditions were tested by the combination of four IPTG concentrations (0.25, 0.5, 0.75, and 1.0 mM), four Ni^2+^ concentrations (0.3, 0.5, 0.75, and 1.0) and incubation at six temperatures (18, 20, 22, 25, 27, and 30°C) for various periods of time (18, 24 and 30 h). The cells were grown with intensive aeration (180 rpm). Next, 15-mL aliquots of the culture were harvested via centrifugation (16 500 x g, 30 min with cooling). The cell pellets were washed twice with an equal volume of ice-cold phosphate buffer (50 mM KH_2_PO_4_, 50 mM KHPO_4_, and 50 mM Na_2_SO_3_, pH 7.5) that contained four different Ni^2+^ concentrations and 0.1 mM EDTA and were then resuspended in 2 mL of the same buffer solution. The cells were disrupted using a Cole Parmer Torbeo 36800 600-W sonicator. The cell debris was separated via centrifugation at 16 500 x g for 30 min at 4°C. The extract was additionally filtered using a 0.45-μm pore membrane (Millex GV Millipore) and assayed for urease activity using the phenol-hypochlorite ammonia quantification method [30]. Protein concentration was measured using the method of Bradford [31]. The cell-free supernatant and insoluble protein aggregates were analyzed via SDS-PAGE electrophoresis to determine the effects of temperature and inducer concentration on the production and solubility of the protein. All experiments were performed in triplicate.

Purification of the recombinant enzyme is described in the S1 File.

### Enzyme and inhibition studies

The kinetic parameters of urease were determined by measuring the initial rates of the reactions in 50 mM phosphate buffer (pH 7.5) at 37°C. Ammonia was routinely quantified by the phenol hypochlorite method as reported previously [23].

Inhibition studies were conducted by initiating the enzymatic reaction with the addition of the enzyme into assay mixtures (200 μL total volume) containing increasing concentrations of inhibitors and 0.1–2.5 mM urea. After 15 min of incubation at 37°C, the reaction was stopped by adding phenol-hypochlorite reagents and the absorbance of the formed indophenol blue complexes was measured at 650 nm. The *K*_*i*_ values were determined from Lineweaver-Burk plots after testing at least five inhibitor concentrations in a range that depended on their inhibitory strength. The concentration of inhibitor that caused the loss of 50% of the enzyme’s activity (*IC*_50_) was determined from the measurements that were performed at a urea concentration of 0.6 mM and calculated using the linear regression of the urease activity versus the logarithm of the inhibitor concentrations.

The values of *K*_*i*_ and *IC*_50_ for all analyzed compounds were calculated using the appropriate equations in GraphPad Prism 5. Errors were reported with two significant digits, and each measured parameter was given with the same number of decimal places as its error [32].

### Urease inhibition in whole cells of *E*. *coli* + pGEM::ureOP

The expression strain *E*. *coli* + *pGEM*::*ureOP* was cultured in 150 mL of LB broth (Biocorp, Poland) with 100 μg/mL ampicillin (Polfa, Tarchomin, Poland) at 37°C and induced with IPTG (0.75 mM) and Ni^2+^ (0.75 mM) during the exponential growth phase, which was determined by the OD_600_ measurements (OD_600_ = 0.6). Further expression was conducted for 24 h at 22°C. The bacterial culture (1 mL) was harvested via centrifugation (2 min, 20 376 x g, 37°C) and washed three times with PBS. The pellets from 1-mL samples of the expression cell culture were resuspended in 100 μL of PBS. The hydrolysis of urea that is catalyzed by whole bacterial cells was performed in 96-well plates (Rotilabo, U-profile). The reaction progress curves were obtained by the addition of bacterial cells (1×10^9^ CFU/mL final concentration) into mixtures (300 μL final volume) that contained 5 mM urea and various inhibitor concentrations that depended on their inhibitory strength. Alternatively, the reaction was initiated via the addition of a concentrated solution of urea after a 120-min preincubation of bacterial cells with the inhibitor. Samples (50 μL) of the reaction mixture were removed after the appropriate reaction time. The enzymatic reactions in the samples were terminated via the introduction of 100 μL of phenol-sodium nitroprusside solution, immediately followed by the addition of 100 μL of NaOH-hypochlorite solution. The absorption of indophenol blue was measured at 650 nm using a TECAN-Sunrise absorbance reader equipped with a gradient filter and Magellan software [30]. The *IC*_50_ values were determined from the linear regression of the urease activity versus the logarithm of the inhibitor concentration. The reaction was studied in the absence and in the presence of the inhibitor. The influence of dimethylformamide (DMF), which was used as a solvent for several compounds, was also determined.

### *H*. *pylori* J99 growth conditions

Reference strain J99 that had been stored in tryptic soy broth (TSB) medium (Oxoid) supplemented with 15% glycerol at -70°C was revived and plated on two kinds of culture media: Columbia agar (from Difco) supplemented with 7% hemolysed horse blood and selective Columbia agar medium supplemented with 7% hemolysed horse blood and enriched with a selective supplement (Oxoid company) composed of 10 mg/L vancomycin, 10 mg/L trimethoprim, 5 mg/L cefsulodin, and 5 mg/L amphotericin B. The cultures were incubated for 3 days under microaerophilic conditions (5% O_2_, 10% CO_2_, 85% N_2_) at 37°C, subcultured onto fresh Columbia agar media and incubated 3 more days under the same conditions.

To obtain a broth culture, 72-h cultures of the *H*. *pylori* reference strain were used. Then, each isolate was harvested using a sterile swab from one dish of the Columbia agar medium and passaged to 10 mL of Brucella broth (Oxoid) (containing 5% fetal bovine serum (Sigma) and 1% IsoVitalex (from BBL)). If needed, 15 mM urea and 0.05 μM nickel(II) chloride (Carl Roth) were added to the growth medium. The cultures were grown at 37°C for 48 h with vigorous shaking and under microaerophilic conditions in anaerobic jars using Genbox microaer kits (bioMerieux).

### Native *H*. *pylori* urease determination

The *H*. *pylori* strain was passaged and cultured as described above. An aliquot of the culture (3 mL) was harvested via centrifugation (16 500 x g, 5 min, 10°C). The cell pellets were washed twice with an equal volume of ice-cold phosphate buffer (50 mM KH_2_PO_4_, 50 mM K_2_HPO_4_, and 50 mM Na_2_SO_3_, pH 7.5) and disrupted using a Cole Parmer Torbeo 36800 600-W sonicator. The cell debris was separated via centrifugation at 16 500 x g for 5 min at 10°C. The lysate was filtered through 0.45-μM PVDF filters and then applied to a Phenyl Sepharose XK 26/20 GE Healthcare hydrophobic interaction column that was equilibrated with buffer A containing 1 M KCl. Urease was eluted with a descending linear gradient of KCl (from 1 M to 0 M) in buffer A. Samples of soluble protein (from lysate and after sonication) were subjected to SDS-PAGE.

### Urease inhibition in whole cells of *H*. *pylori* J99

Bacterial cells were harvested by centrifugation and washed with 10 mM PBS, pH 7.2, as high concentrations of albumin cause unstable indophenol blue formation in the ammonia quantification reaction. Additional washing steps were avoided as they could potentially eliminate the membrane-integrated enzyme from the test suspensions. The washed cells were resuspended in PBS to correspond to the 0.5 McFarland standard (approximately 1–2×10^8^ CFU/mL). Reaction mixtures contained a 10-fold dilution of prepared bacterial mixture in PBS, 10 mM urea, and chosen concentrations of examined urease inhibitors. The reaction mixtures were incubated at 37°C in an ELMI DTS-4 SkyLine orbital shaker. Urease activity was routinely determined by monitoring ammonia ion formation, as described above. When needed, *H*. *pylori* cells were preincubated for 2 h under microaerobic conditions with the inhibitory compounds in their growth medium. After preincubation, the bacteria were washed with PBS and treated in the same manner as non-preincubated cells.

### Antiproliferative activity of urease inhibitors

#### Cell culture

BALB/3T3, MCF-10A, and Eph4-Ev cell lines were purchased from the American Type Culture Collection (ATCC Rockville, Maryland, USA). An HECa10 cell line was obtained from the Institute of Immunology and Experimental Therapy and was established by Dr M. Paprocka and Prof. C. Kieda as previously described [33]. Cell lines were maintained at the Institute of Immunology and Experimental Therapy (IIET), Wroclaw, Poland.

The BALB/3T3 cell line was cultured in Dulbecco MEM (Gibco, Scotland) supplemented with 10% (v/v) FBS (Sigma-Aldrich, Poznań, Poland) and 2 mM L-glutamine (Sigma-Aldrich, Poznań, Poland). The MCF-10A cell line was cultured in F-12 Nutrient mixture supplemented with 5% (v/v) horse serum (both Gibco, Scotland), 0.05 μg/mL cholera toxin, 10 μg/mL insulin, 0.5 μg/mL hydrocortisone, and 20 ng/mL EGFH (all Sigma-Aldrich, Poznań, Poland). The Eph4-Ev cell line was cultured in Dulbecco MEM supplemented with 2 mM L-glutamine, 10% (v/v) calf bovine serum (ATCC Rockville, Maryland, USA) and puromycin (Sigma-Aldrich, Poznań, Poland). The HEC A10 cell line was cultured in OPTI-MEM (IIET, Wroclaw, Poland) supplemented with 2 mM L-glutamine and 5% (v/v) FBS. All culture media were additionally supplemented with antibiotics: 100 U/mL penicillin and 100 μg/mL streptomycin (both Polfa-Tarchomin, Poland). During all experiments, cells were maintained in a humid atmosphere at 37°C and 5% CO_2_ and passaged twice a week using EDTA-Trypsin (pH 8; IIET, Wroclaw, Poland) solution as a detachment agent.

#### Sulforhodamine B antiproliferative assay

This assay was used as previously described [34] with minor modifications. Briefly, 24 h before adding the tested compounds, cells were seeded in 96-well plates (Sarstedt, Germany) in appropriate culture medium with 10^5^ cells/mL cells/mL (100 μL/well). Cells were treated with each compound in at least four concentrations in the range from 500–4 μM for 72 h. Next, cells were fixed with 50 μL/well of 50% (w/v) trichloroacetic acid (Avantor Performance Materials, Gliwice, Poland). After a 1-hour incubation, the plates were washed several times with tap water, and 50 μL of 0.4% (w/v) solution of sulforhodamine B (Sigma-Aldrich, Germany) in 1% (v/v) acetic acid (Avantor Performance Materials, Gliwice, Poland) was added to each well. After a 30-min incubation at room temperature, unbound dye was washed out with 1% (v/v) acetic acid, whereas bound dye was solubilized with 10 mM unbuffered TRIS (Avantor Performance Materials, Gliwice, Poland) solution. The entire procedure was performed using an EL-406 washing station (BioTek Instruments, USA). Next, absorbance at 540 nm was measured using a Synergy H4 Hybrid Reader (BioTek Instruments, USA). Compounds at each concentration were tested in triplicate in a single experiment, and each experiment was repeated three times independently. The results were calculated using the Prolab-3 system based on Cheburator 0.4 software [35] and was presented as proliferation inhibition percentages at the highest concentration used.

### Statistical analysis

The results concerning the inhibitory efficiency of studied compounds were analyzed using GraphPad Prism 7.02 (GraphPad Software, LaJolla California USA) utilizing statistical methods indicated under tables.

## Results and discussion

### Optimization of *H*. *pylori* urease expression

*H*. *pylori* urease, a high-molecular-mass (550 kDa) multimeric enzyme, is encoded by two subunit genes, *ureA* and *ureB*. These genes alone are sufficient to encode a fully assembled but catalytically inactive apoenzyme that undergoes maturation in a stepwise assembly process with the participation of four accessory proteins (UreD, UreF, UreG, and UreE), leading to the nickel-loaded active holoenzyme [28]. For these reasons, it had been very difficult to secure high-level recombinant *H*. *pylori* urease activity in *Escherichia coli* strains [36, 37]. Mobley et al., when using an *E*. *coli* SE5000 strain transformed with a 15.3-kbp plasmid (pHP8080) that encoded both *H*. *pylori* urease and the NixA nickel transporter, obtained recombinant urease with good catalytic activity. This plasmid was created by the insertion of a fragment, much longer than the urease operon, that was generated by a random restriction of the *H*. *pylori* genome that conferred a urease-positive phenotype to *E*. *coli* cells. The details of the inserted sequence are not available. In addition, pHP8080 does not contain an *E*. *coli* promoter upstream of the operon; therefore, transcription in *E*. *coli* must rely on the presence of a promoter from *H*. *pylori* urease genes, which may be recognized less efficiently by *E*. *coli* RNA polymerase [38]. Herein, plasmid *pGEM*::*ureOP*, which encodes the entire urease gene cluster, was used to produce recombinant *H*. *pylori* urease [28]. The urease operon was recloned from the genome of *H*. *pylori* G27 using PCR, and the PCR product was inserted into the pGEM-T Easy vector (Promega). This recombinant plasmid has the urease operon in the same transcriptional orientation as the T7 promoter, allowing urease expression to be induced by IPTG when the bacteria are grown in a medium containing nickel ions [28].

**Fig 1:**
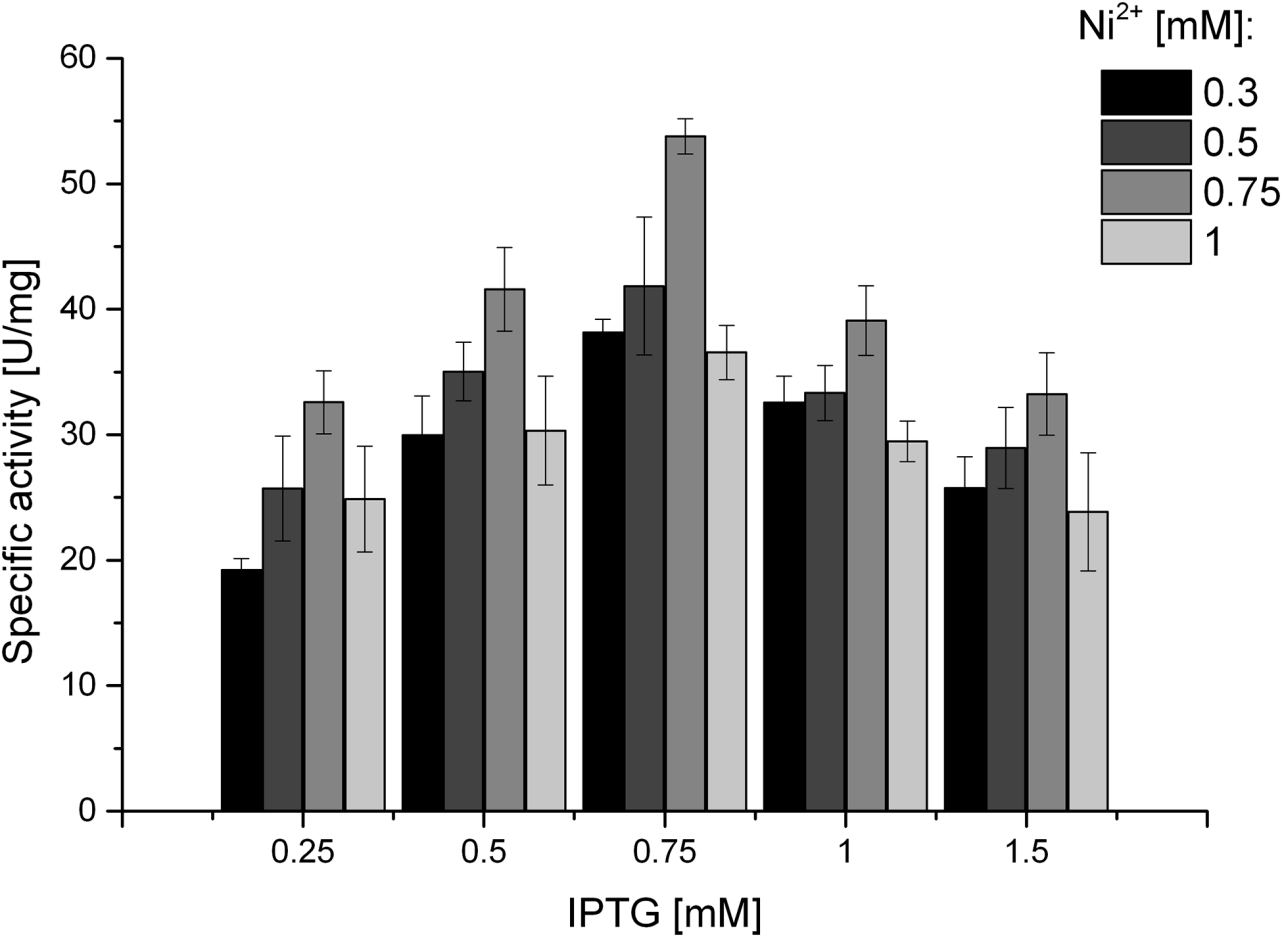
The influence of IPTG and nickel ion concentration on the expression of recombinant *H*. *pylori* urease in *E*. *coli* Rosetta2 (DE3) strain.

**Fig 2:**
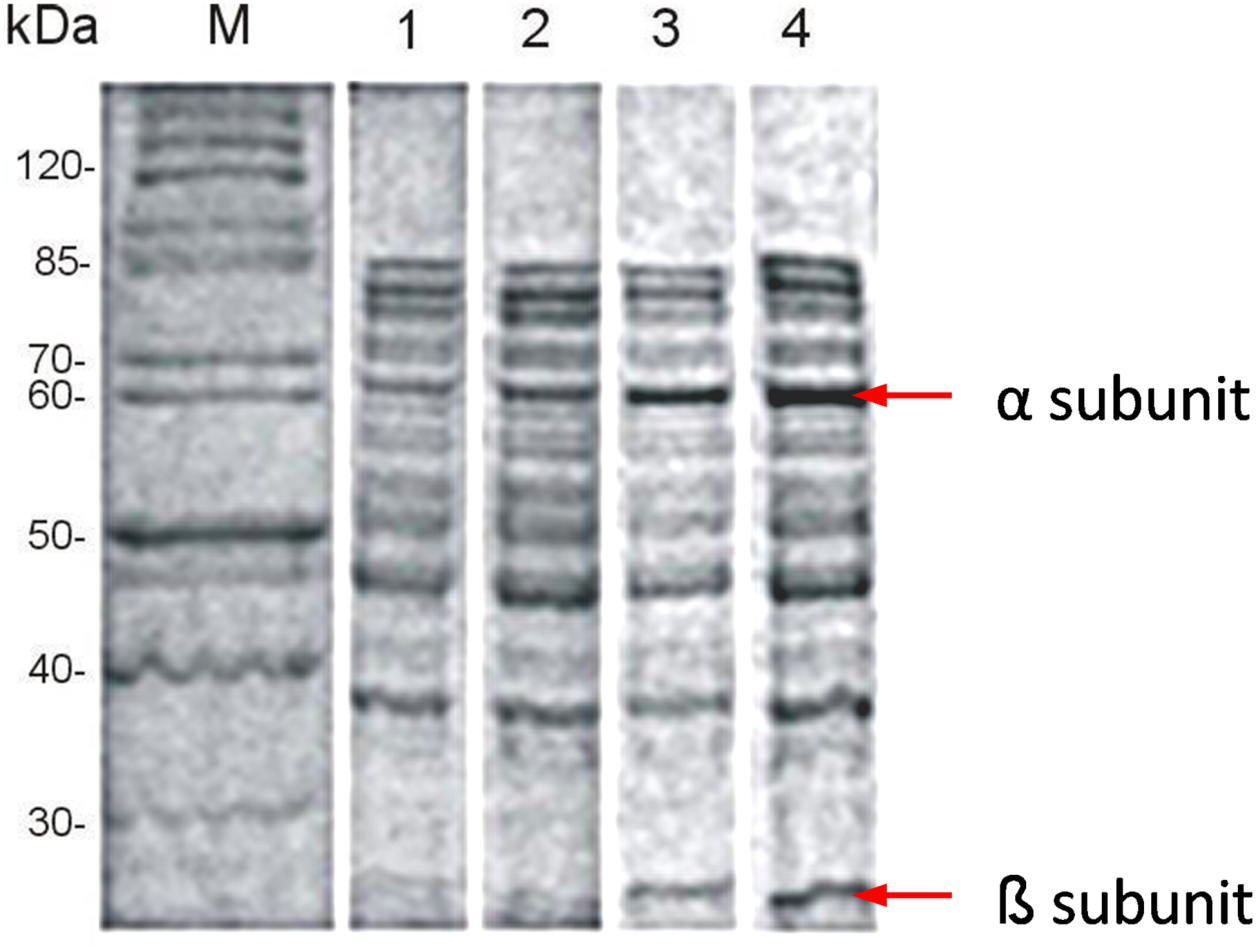
The optimization of the induction conditions for the highly efficient expression of soluble protein. *E*. *coli* Rosetta2 (DE3) that contained *pGEM*::*ureOP* was grown at 37°C. When the OD_600_ reached 0.6, protein expression was induced under 96 different conditions. The cells were grown for an additional 18 h, harvested, and disrupted via sonication, and analyzed using SDS-PAGE electrophorograms. Lane M: protein molecular weight marker (kDa). Lane M: protein molecular weight marker (kDa). Cell-free extracts obtained from cultures carried out at different steps of optimization. Line 1: 750 μM Ni ^2+^; Line 2: 750 μM IPTG, and 750 μM Ni^2+^; Lane 3: induction temperature 22°C, 750 μM IPTG and 750 μM Ni^2+^; Line 4: time of incubation 24 h, 22°C, 750 μM IPTG and 750 μM Ni^2+^.

The massive overexpression of one particular protein usually requires optimization of specific expression conditions. In the first step, the effects of the IPTG inducer and the nickel ion concentration on the productivity and solubility of the protein were determined. It has been established that growth temperature has a significant effect on the production and folding of heterologous proteins [39], and therefore, the influences of temperature changes in the range from 18 to 30°C and the time of incubation on urease expression were tested. After disintegration, the cell-free supernatant was analyzed using a Berthelot spectrophotometric urease assay [30] and SDS-PAGE. The expression level was notably high in the presence of 750 μM IPTG and 750 μM Ni^2+^ (Fig 1). The optimal temperature was 22°C. We found that reducing the growth temperatures of cells increased the solubility of recombinant proteins, in agreement with known dependencies. Furthermore, the presence of 1 mM EDTA in 50 mM HEPES buffer (pH 7.5) beneficially contributed to improving the stability of urease preparations. The progress of the optimization procedure was also monitored using SDS-PAGE electrophorograms (Fig 2) and was confirmed by the results of specific activity measurements.

### Inhibition of purified urease from *H*. *pylori*

**Fig 3:**
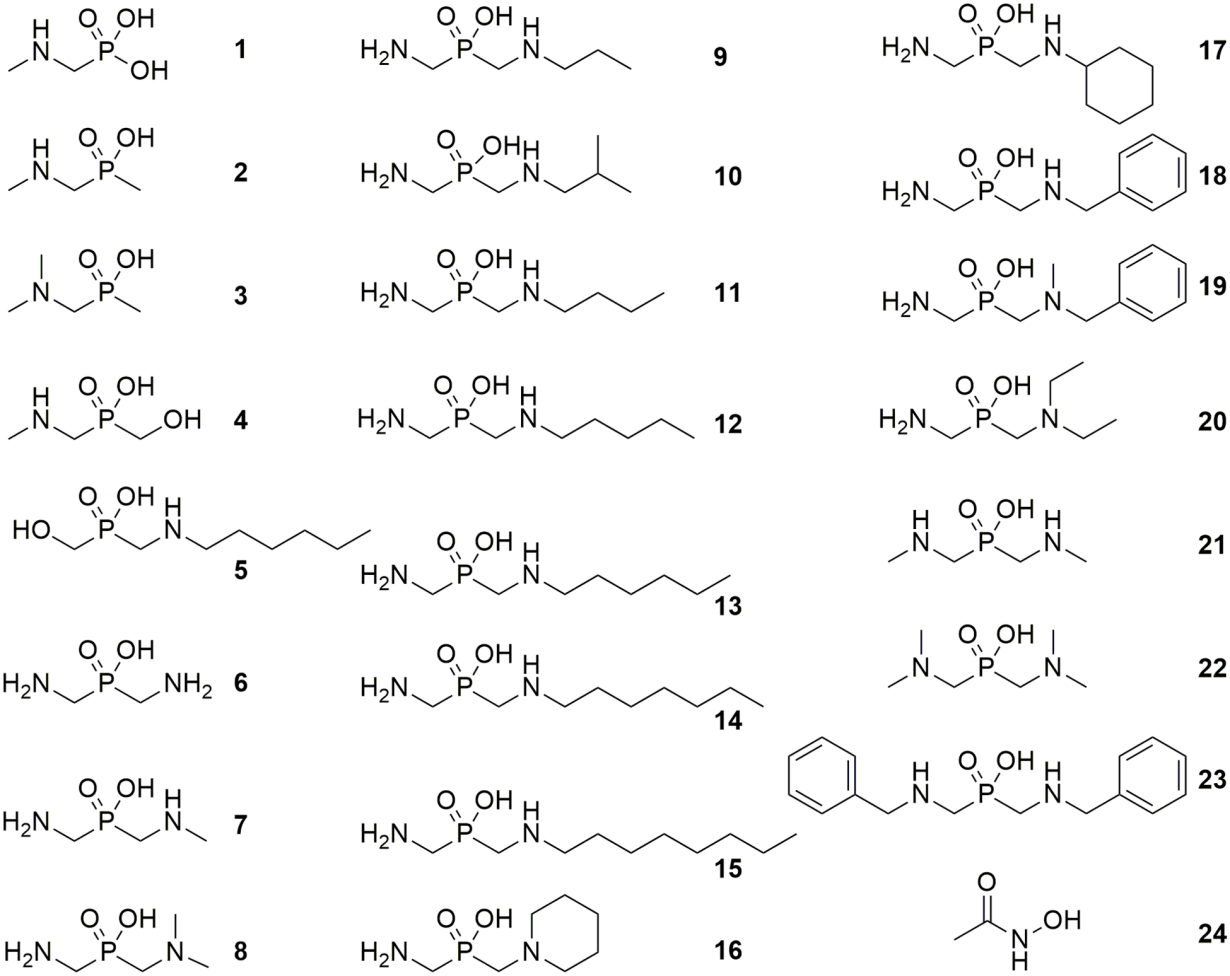
Structures and numbering of phosphinic and phosphonic compounds tested for inhibition of recombinant urease from *H*. *pylori*.

The progress curves of the urease reaction and the kinetic analysis of the inhibition in the presence of increasing concentrations of urea determined that the assayed derivatives are competitive reversible inhibitors with inhibition constants (*K*_*i*_) in a micromolar range (for most of the compounds, S1 Table). Initially, a comparison of four compounds (**1–4**, Fig 3) that represent aminomethylphosphonates, aminomethyl-*P*-methylphosphinates and aminomethyl-*P*-hydroxymethylphosphinates groups, which had been shown to be the most active against *S*. *pasteurii* and *P*. *mirabilis* ureases [23–27], was performed. The observed pattern of *H*. *pylori* urease activity inhibition was similar to those observed for the other abovementioned bacterial ureases, and *N*-methylaminomethyl-*P*-hydroxymethylphosphonic acid (**4**) exhibited the highest inhibitory activity within this group, with *K*_*i*_ = 1.03 μM (Table 1).

**Table 1:** Inhibitory activities (*K*_*i*_ values) of phosphinic and phosphonic compounds against purified recombinant urease from *H*. *pylori*.

no	*K*_i_ [μM]	no	*K*_i_[μM]	no	*K*_i_[μM]
**1**	38.3 ± 1.1*	**9**	22.1 ± 2.1	**17**	NI
**2**	61.6 ± 3.5*	**10**	440 ± 70*	**18**	44.4 ± 2.5*
**3**	9.27 ± 0.35*	**11**	27.0 ± 2.1	**19**	36.4 ± 4.9*
**4**	1.032 ± 0.068*	**12**	43.1± 3.6*	**20**	NI
**5**	74.3 ± 4.7*	**13**	0.294 ± 0.013*	**21**	26.1 ± 1.8
**6**	61.0 ± 9.2*	**14**	878 ± 25*	**22**	NI
**7**	20.9 ± 2.0	**15**	NI	**23**	50.7 ± 4.8*
**8**	29.9 ± 2.0	**16**	NI	**24**	23.2 ± 1.5

* p< 0.01 to **24**, one-way ANOVA with Dunnett’s multiple comparison post-test with **24** indicated as a reference compound

Subsequently, the group of bis(aminomethyl)phosphinic acid derivatives was studied in more detail. Compounds containing symmetrically and unsymmetrically distributed single methyl substituents (**7**, **8** and **21**) were characterized with at least similar and in most instances higher activity than the unsubstituted compound **6**. The incorporation of additional methyl groups into inhibitor **21**, yielding the tetramethylated compound **22**, resulted in a loss of inhibition. This was likely due to a deficiency of two hydrogen bonds in the inhibitor enzyme. The structure of active sites of ureases from different sources is highly conserved. Therefore, the pattern of the hydrogen bond network of the inhibitor amine group that was predicted by molecular modeling of the inhibitor **13**−*S*. *pasteurii* urease complex was in good agreement with the experimentally obtained affinities [27]. The increase in substituent size from a methyl (**21**) to benzyl group (**23**) contributed to a decrease in the inhibitor affinity for the enzyme.

The compounds with one to five carbon atom linear extensions of the substituent chain (**7**, **9**, **11** and **12**) exhibited average activity in the range from 20.9–43.1 μM. The inhibition constant of the most active compound in this series and among all inhibitors that contain a C-N-P core, the *n*-hexyl derivative **13**, was 0.29 μM. Interestingly, replacement of one amino group in **13** with the hydroxyl group in **5** resulted in a decrease in activity by more than 2 orders of magnitude. A dramatic loss in potency was also observed for phosphinates containing an *n*-heptyl (**14**) or *n*-octyl (**15**) substituent. The increase in the steric hindrance of one amino group in bis(aminomethyl)phosphinic acid led to a decrease (**10**) or even complete deficiency in inhibitory activity (**16**, **17** and **20**).

Recombinant *H*. *pylori* urease was generally less susceptible, although still significantly inhibited, by the assayed compounds and exhibited a nearly identical pattern of the structure-activity relationship as other bacterial ureases that were described previously [23–25, 27]. Statistical analysis of results indicated three compounds (namely **3**, **4** and **13**) as significantly more potent in comparison to acetohydroxamic acid (**24**).

### Ureolytic activity of *H*. *pylori* J99

**Fig 4:**
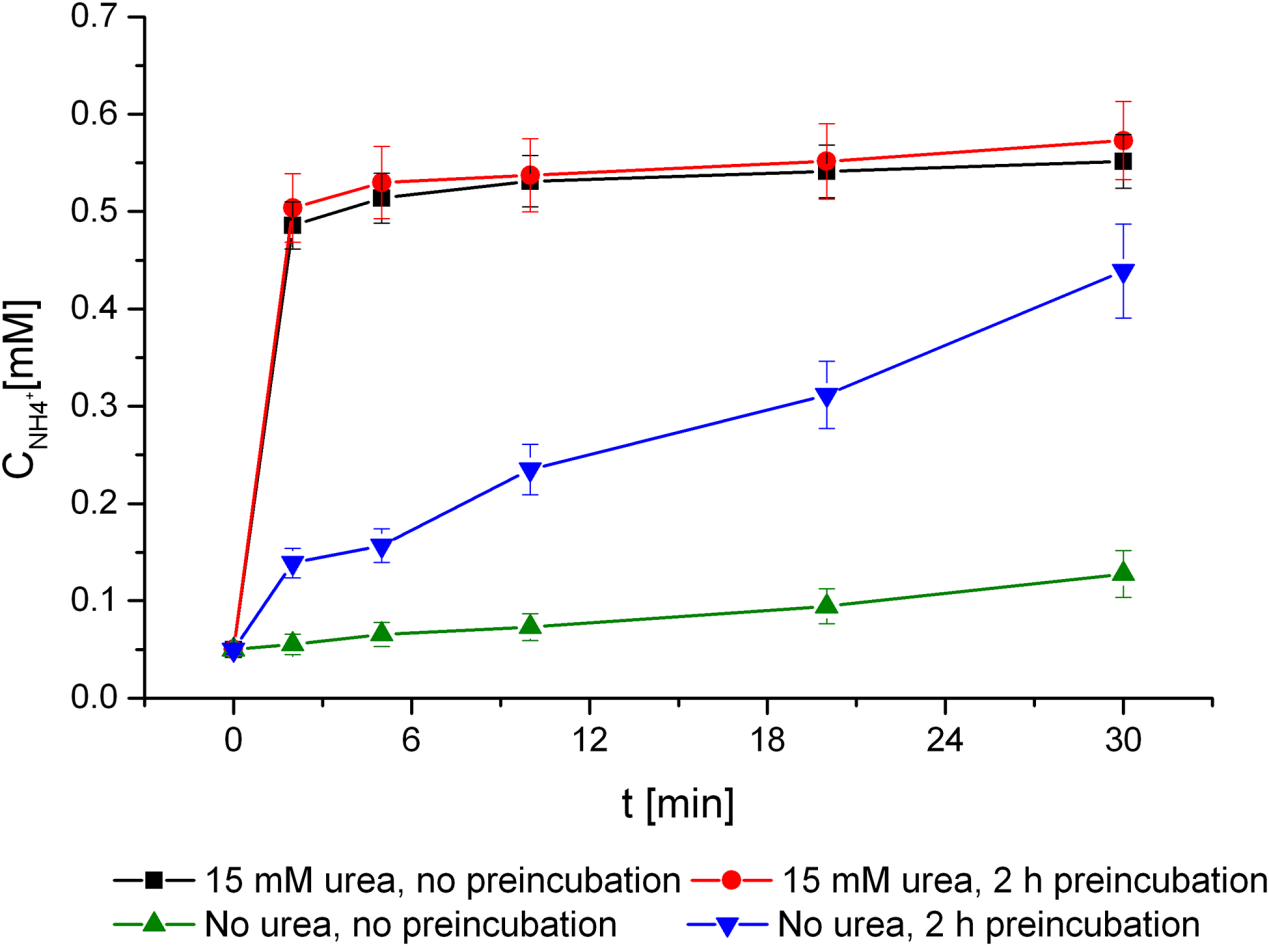
Ammonia release due to urease activity of *H*. *pylori* J99. Cells grown for 24 h in the presence or absence of urea, assayed directly after harvesting or after 2 h of incubation in PBS under microaerobic conditions. No inhibitors were present.

The activity of native urease that was detected in intact *H*. *pylori* J99 cells depended strongly on the growth conditions and applied assay method. Cells cultured in the presence of 15 mM urea for 48 h exhibited a urease burst upon incorporation into the assay mixture, after which the enzyme activity soon reached a low plateau (Fig 4). The rate of product release in the burst phase was 8.3–8.7 nmol/min/1.5×10^7^ CFU, which then quickly decreased to 0.05–0.07 nmol/min/1.5×10^7^ CFU. Attempts to calibrate the assay so that it remained in a linear range failed for urea-grown bacteria. Whether they were harvested and immediately used in the urease assay or preincubated with inhibitory compounds for 2 h (either in original growth medium or in urea-free PBS) under microaerobic conditions, the ureolytic activity profiles were the same after the catalyst was introduced into the reaction mixture. This behavior occurred in both untreated cells (Fig 4) and in assays containing urease inhibitors in concentrations that reduced enzyme activity by more than 70%.

**Fig 5:**
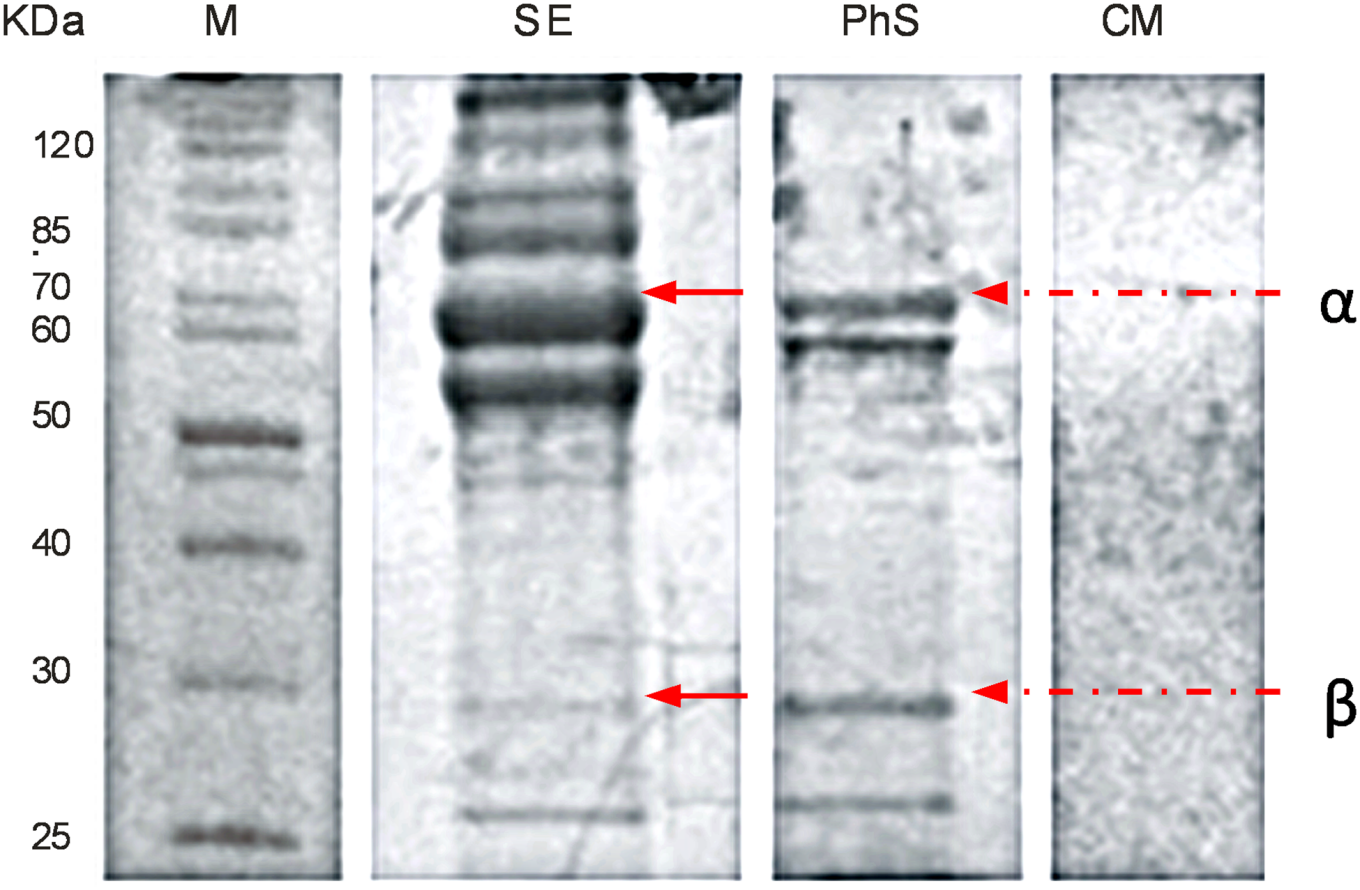
SDS-PAGE electrophorograms of native *H*. *pylori* J99 urease partially purified from the culture supernatant. Cells were grown for 72 h in the presence of 15 mM urea. M–molecular weight marker, SE–a soluble protein fraction before purification, PhS–fractions containing active urease after hydrophobic interaction purification using Phenyl Sepharose, CM–original culture medium (control). The red arrows indicate α and β subunits of urease with molecular weights of 61.7 and 29.5 kDa, respectively.

Urease inhibitors reduced the intensity of the burst phase in a dose-dependent manner in each assay. Thus, the ammonia that was measured in the assays was truly a product of active *H*. *pylori* urease and not of excess ions that were released from the periplasmic space of cells injured during the harvest or assay procedure. When *H*. *pylori* J99 strain was grown with urea for 3 days, the medium pH was elevated to 8.5 and a significant amount of urease could be detected in the culture medium after cell separation. The presence of free enzyme was confirmed by SDS-PAGE electrophoresis (Fig 5). When the urea-grown cultures were cultured for 48 h, all detectable urease activity was associated exclusively with bacterial cells, and such growth conditions were employed in the experiments involving intact microorganisms.

*H*. *pylori* J99 cells grown in Brucella broth without urea exhibited a lower level of constitutively expressed urease (0.10 nmol/min/1.5×10^7^ CFU). After harvesting and transferring the cells into the urease assay that contained 5 mM urea, the amount of product per minute in the uninhibited reaction mixtures rose to 0.17 nmol/min/1.5×10^7^ CFU during a 30-min incubation. When cells were additionally subjected to a 2-h preincubation in urea-free PBS buffer, the urease activity that was detected at the initial assay time was 0.30 nmol/min/1.5×10^7^ CFU; this activity increased quickly in uninhibited controls to 0.63 nmol/min/1.5×10^7^ CFU during the 30-min reaction in 5 mM urea (Fig 4). The induction of urease was most likely a response to nitrogen starvation during the preincubation step and was then further enhanced due to exposure to the high concentration of urea during the assay.

It was clear that the overall urease level in intact *H*. *pylori* cells not only depended on the presence or absence of urea during growth but also shifted during the activity measurements in the presence of substrate. This change, of course, affected the results of the inhibition determination in the reactions that contained the tested compounds. We also had to consider that the precise evaluation of inhibitor efficiency in a whole-cell system is even more complicated due to the dual locations of the *H*. *pylori* enzyme (cytoplasmic and in a surface-bound form, in an unknown ratio).

### Inhibition of urease in transformed *E*. *coli*

**Fig 6:**
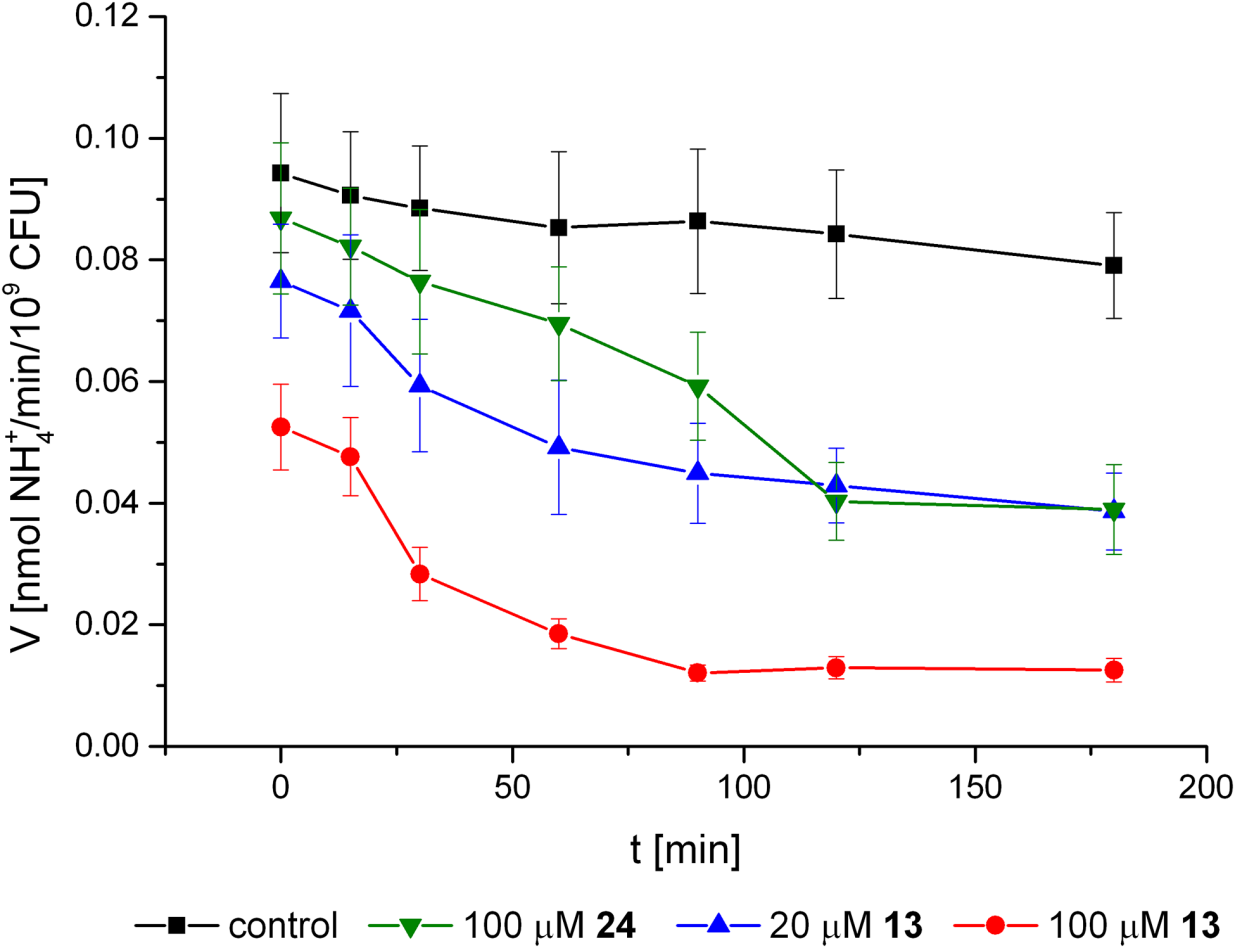
Inhibitory activity of compounds 24 (acetohydroxamic acid) and 13 (*N*-*n*-hexylaminomethyl-*P*-aminomethylphosphinic acid) against urease in intact *E*. *coli* (*pGEM*::*ureOP*) cells.

For the proximate determination of the inhibitor’s cell permeability, *E*. *coli* (*pGEM*::*ureOP*) served as a model of a gram-negative microorganism that contained *H*. *pylori* urease only inside the cell. The activity of intracellular recombinant urease in intact recipient cells in PBS buffer was stable even in the progress curves that were recorded for 3 h (Fig 6).

The degree of urease inhibition in *E*. *coli*, expressed by the IC_50_ parameter and calculated from 30-min reactions that lacked a preincubation with inhibitors, ranged from approximately 150 μM to above 2.5 mM (Table 2, $IC_{50}^{0}$). A complete lack of inhibitory effects was observed for compounds **6**, **14, 18**, **19** and **23**, which could be classified as moderate inhibitors of purified recombinant *H*. *pylori* urease. To simplify the interpretation of the results, inhibitors with an *IC*_50_ exceeding 2 mM were also considered to not be active because of their low biological relevance. The most efficient inhibition of recombinant cytoplasmic urease in non-preincubated assays was exerted by structurally unextended molecules: *N*-methylaminomethylphosphonic acid (**1**), *N*,*N*-dimethylaminomethyl-*P*-methylphosphinic acid (**3**) and acetohydroxamic acid (**24**). Among the derivatives of bis(aminomethyl)phosphinic acid, only the structure bearing a six-carbon chain extension (*N*-*n*-hexylaminomethyl-*P*-aminomethylphosphinic acid (**13**) expressed similar activity, with an *IC*_50_ below 200 μM. This compound had the lowest dissociation constant (*K*_*i*_ = 0.29 μM) toward purified recombinant *H*. *pylori* urease (Table 1). The progress curves of the urease reaction in the presence of inhibitors showed that the degree of inhibition increased with time (Fig 6). The *IC*_50_ parameter after 2 h of incubation with tested compounds (Table 2, $IC_{50}^{2h\;PBS}$) was lower for all active compounds, and the highest activity enhancement was observed for the branched *N*,*N*-dimethyl structure (**3**) and the extended derivative of bis(aminomethyl)phosphinic acid (**13**). Preincubation with the latter compound resulted in a ten-fold growth in efficiency. In the preincubated *E*. *coli* (*pGEM*::*ureOP*) system, the *IC*_50_ values of compounds **3** and **13** were in the low micromolar range (25.1 and 16.9 μM, respectively). Two-way ANOVA analysis of the effects of inhibitor structure and preincubation conditions (Sidak’s multiple comparisons test used as post-test) showed statistically significant increase of the degree of inhibition after preincubation for almost all compounds. Dunnett’s multiple comparisons test used as post-test indicated that none of the tested compounds was statistically significantly more active than acetohydroxamic acid ($IC_{50}^{2h\;PBS}\;=\;96\mu \text{M}$).

**Table 2:** Comparison of inhibitory efficiency against whole cells of transformed *E*. *coli* and *H*. *pylori* J99. $IC_{50}^{0}$ –parameter assayed without preincubation of cells with inhibitory compounds; $IC_{50}^{\text{2h PBS}}$ –parameter assayed using cells subjected to 2 h of preincubation with inhibitors in PBS under microaerobic conditions; $IC_{50}^{2h\;medium}$–parameter assayed using cells subjected to 2 h of preincubation with inhibitors in growth medium under microaerobic conditions.

*E*. *coli* (*pGEM*::*ureOP*)			*H*. *pylori* J99				
			grown with urea			grown without urea	
Compound no	$IC_{50}^{0}$[μM]	$IC_{50}^{2h\;PBS}$[μM]	$IC_{50}^{0}$[μM]	$IC_{50}^{2h\;medium}$[μM]	$IC_{50}^{2h\;PBS}$[μM]	$IC_{50}^{0}$[μM]	$IC_{50}^{2h\;PBS}$[μM]
**1**	164 ± 24	110 ± 21	400.4 ± 8.7^c^	202 ± 43	349 ± 29^c^	316 ± 18	598 ± 81^b^
**2**	573 ± 76	306 ± 48^a^	597.4 ± 5.2^c^	274 ± 87	468 ± 21^c^	480 ± 79	852 ± 40^b^
**3**	179 ± 32	25.1 ± 4.9	640 ± 18^c^	304 ± 16^d^	395 ± 37^c^	530 ± 24	765 ± 31^b^
**4**	759 ± 106	430 ± 63^a^	465 ± 42^c^	207 ± 11	373 ± 48^c^	341 ± 41	676 ± 23^b^
**6**	NI	NI	NI	NI	NI	1302 ± 239	611 ± 63
**7**	880 ± 105	440 ± 61^a^	NI	574 ± 44^d^	456 ± 45^c^	327 ± 68	418 ± 48
**9**	746 ± 94	306 ± 43^a^	1500 ± 160	553 ± 44^d^	940 ± 59^c^	772 ± 143^b^	883 ± 96
**10**	NI	1868 ± 113	NI	835 ± 34^d^	1132 ± 83^c^	913 ± 63	980 ± 35
**11**	779 ± 99	284 ± 41^a^	732.8 ± 9.2^c^	470 ± 38^d^	816 ± 39^c^	881 ± 35	933 ± 206
**12**	1667 ± 232	475 ± 73^a^	813 ± 61^c^	806 ± 72^d^	853 ± 93^c^	872 ± 68	917 ± 105
**13**	186 ± 30	16.9 ± 2.2	510 ± 16^c^	205 ± 15	608 ± 23^c^	597 ± 19	742 ± 55
**14**	NI	NI	942 ± 79^c^	474 ± 34^d^	672 ± 52^c^	743 ± 74^b^	675 ± 33
**15**	NI	NI	1012 ± 84^c^	276 ± 17	712 ± 28^c^	1261 ± 53^b^	638 ± 22
**16**	NI	NI	NI	NI	NI	NI	NI
**17**	NI	NI	NI	NI	NI	NI	NI
**18**	NI	NI	NI	NI	NI	NI	NI
**19**	NI	NI	NI	NI	NI	NI	NI
**20**	NI	NI	NI	NI	NI	NI	NI
**21**	1258 ± 189	269 ± 39^a^	443 ± 24^c^	117.4 ± 7.4	549 ± 15^c^	581 ± 37	132 ± 21^b^
**22**	NI	NI	NI	NI	NI	NI	NI
**23**	NI	NI	NI	NI	NI	NI	NI
**24**	153 ± 31	96 ± 17	1666 ± 70	192 ± 26	1356 ± 194	NI	156 ± 32^b^

^a^ p< 0.05 to the corresponding results; two-way ANOVA with Sidak’s multiple comparisons test post-test

^b^ p<0.05 to the corresponding results; two-way ANOVA with Sidak’s multiple comparisons test post-test

^c^ p <0.01 to 24 under the same growth conditions, two-way ANOVA with Dunnett’s multiple comparison test post-test

^d^ p< 0.01 to 24 under the same growth conditions, one-way ANOVA with Dunnett’s multiple comparison test post-test

### Inhibition of urease in *H*. *pylori* J99

In the intact *H*. *pylori* J99 cells, the urease susceptibility profile was similar to that of *E*. *coli* (*pGEM*::*ureOP*) when cells were grown in the presence of urea and subsequently exposed to inhibitory compounds during preincubation in the original growth medium (Table 2, $IC_{50}^{\text{2h medium}}$). The microplate assays contained calibrated *H*. *pylori* cell concentrations, which ensured that urease reaction rates in the plateau phase were comparable to those of *E*. *coli* (*pGEM*::*ureOP*). The main differences in the calculated *IC*_50_ values were the resistances to compounds **3** and **13**, which were higher by an order of magnitude (25.1 versus 304 and 16.9 versus 205 μM, respectively). The inhibition by acetohydroxamate (**24**) was less efficient by a factor of two (96 versus 192 μM). This reference inhibitor effect was even weaker in urea-grown cells that were assayed for urease without former preincubation and when the preincubation with AHA was conducted in PBS ($IC_{50}^{0}\;=1.66\text{ mM}$ and $IC_{50}^{\text{2h PBS}}=\;1.35\text{ mM, respectively}$).

**Fig 7:**
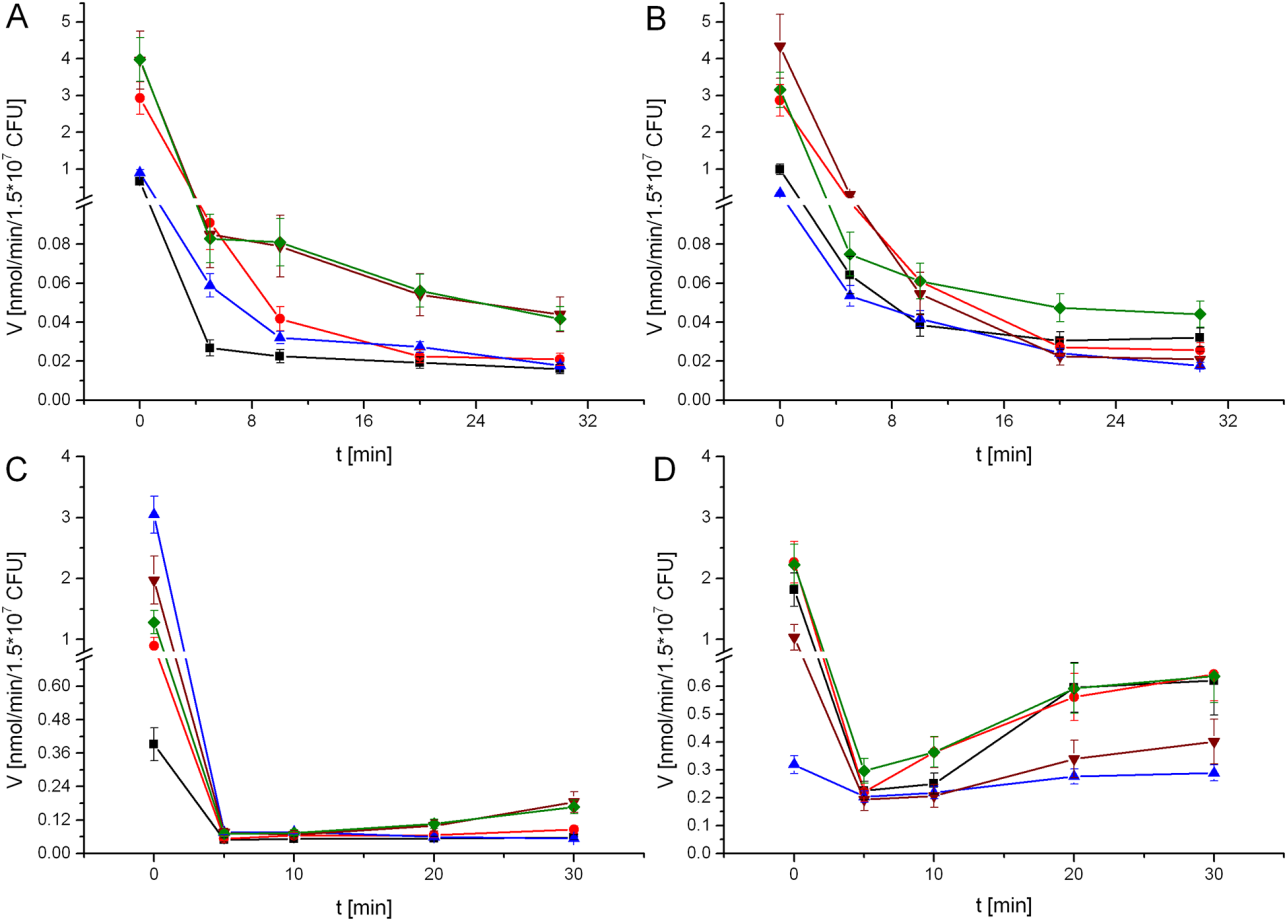
Inhibitory effects of compounds (■) 1, (●) 13, (▲) 21 and (▼) 24 towards urease in intact *H*. *pylori* J99 (♦). Cells grown in the presence of 15 mM urea assayed with 0.5 mM inhibitors without preincubation (A) and after 2 h of preincubation with 0.1 mM inhibitors in the growth medium (B); cells grown without urea assayed with 0.5 mM compounds without preincubation (C) and after 2 h of preincubation with 0.1 mM inhibitors in PBS (D).

The urease activity of cells grown without urea was rapidly induced upon transfer to the urease reaction mixture (Fig 4), especially after preincubation in nitrogen-depleted PBS. Under these last conditions, the effects of the tested reversible inhibitors gradually diminished with the duration of the urease assay. At concentrations ranging up to double the previously estimated *IC*_50_ for urea-grown cells, the inhibitory effect became negligible in most cases within 20 min due to the incorporation of 5 mM urea (see the time course of inhibition by compounds **1** and **13** in Fig 7, panel D). Four compounds, however, behaved in a manner contrary to these others. Acetohydroxamic acid, though not categorized as active in the non-preincubated assay, exhibited significant activity (*IC*_50_ of 156 ± 32 μM) after 2 h of preincubation with the *H*. *pylori* cells. Bis(aminomethyl)phosphinic acid (**6**), inactive in whole-cell systems of Rosetta host *E*. *coli* and urea-grown *H*. *pylori* J99, exerted a moderate inhibitory effect (*IC*_50_ = 1302 ± 239 μM), which was enhanced by preincubation (*IC*_50_ = 611 ± 63 μM). Activity of its single methyl-substituted derivative (**7)** was also higher than that in previous experiments, with *IC*_50_ values below 0.5 mM toward preincubated cells, making it the most potent compound of the series of derivatives **1**–**14**. Finally, bis(*N*-methylaminomethyl)phosphinic acid (**21**) inhibited *H*. *pylori* urease in the preincubated assay (*IC*_50_ of 132 ± 21 μM) the most strongly of all the tested compounds. It should be noted that inhibitor **21** was also the most potent in assays containing *H*. *pylori* J99 cells that were grown in the presence of urea and preincubated with inhibitory compounds in the growth medium (*IC*_50_ = 117.4 ± 7.4 μM).

Statistically significant interaction was observed when inhibitor type and urea presence in the growth medium were indicated as the factors. When the results obtained with or w/o urea in non-preincubated assays ($IC_{50}^{0}$) were compared, statistical differences were observed for **9**, **14**, **15**. When the results obtained with or w/o urea after preincubation in PBS ($IC_{50}^{2h\;PBS}$) were compared, statistical differences were observed for **1**, **2**, **3**, **4**, **21** and **24**. Additionally, when the cells in the urease assays were from urea-grown culture, large number of compounds were statistically more active than **24** (indicated by *c* in Table 2) without preincubation and after preincubation in PBS.

### Cytotoxicity of urease inhibitors

No substantial growth inhibition was observed *in vitro* in sulforhodamine B antiproliferative assays after normal cells (including BALB/3T3 murine cell line, commonly used in *in vitro* cytotoxicity assessment) were treated with the tested compounds, even at concentrations as high as 0.5 mM (Table 3). Inhibitors chosen for cytotoxicity assessment reflected the structural diversity of the studied group and included compounds that were most active against purified and whole-cell urease.

**Table 3:** Cytotoxicity of nine urease inhibitors expressed as inhibition of proliferation in four normal cell lines. Cell proliferation was evaluated using a sulforhodamine B assay against mouse fibroblasts BALB/3T3, murine mammary gland epithelium Eph4-Ev, immortalized murine endothelial cell line from peripheral lymph node HEC A10, and human mammary gland MCF-10A.

% Proliferation inhibition ± SD				
Compound [0.5 mM]	BALB/ƷTƷ	Eph4-Ev	HEC A10	MCF-10A
**1**	2.7 ± 1.8	1.3 ± 2.3	0	0
**2**	1.4 ± 1.1	1.1 ± 1.0	0	0
**4**	0	0	0	0
**6**	3.2 ± 1.6	0	7.7 ± 2.4	0
**7**	0	3.9 ± 2.1	0	0
**9**	0.7 ± 0.6	0	2.2 ± 1.5	0
**13**	6.5 ± 3.6	0	0	0.5 ± 0.4
**15**	0	2.7 ± 1.1	0	1.8 ± 1.2
**21**	0	2.2 ± 1.5	0	0

## Summary

This study represents the first systematic evaluation of the capability of aminophosphonic inhibitors to affect the activity of *Helicobacter pylori* urease. The investigated compounds encompassed three groups of chemical structures that were based on aminomethyl-*P*-methylphosphinic acid, aminomethyl-*P*-hydroxymethylphosphinic acid and bis(aminomethyl)phosphinic acid. This selection reflected the graduate development of a new class of urease inhibitors that was accomplished over the last few years using computer-aided design, refined chemical synthesis and biochemical studies [23–27]. The entire class was intended to be structurally similar to powerful urease deactivators—phosphoramidates—but, in contrast to these molecules, members of the new class were hydrolytically stable due to the presence of a N-C-P scaffold instead of the labile N-P linkage. In previous reports, the interaction of the inhibitors with purified urease from *Sporosarcina pasteurii* (SPU) and *Proteus mirabilis* (PMU) was investigated. That past research noted that *N*,*N*-dimethylaminomethyl-*P*-aminomethylphosphinic acid (**3**), *N*-methylaminomethyl-*P*-hydroxymethylphosphinic acid (**4**), and *N*-*n*-hexylaminomethyl-*P*-aminomethylphosphinic acid (**13**) were the most effective members of the group and acted as competitive reversible inhibitors of bacterial ureases. Their dissociation constants were in the submicromolar range (**3**, *K*_*i*_ = 0.62 μM against SPU; **4**, *K*_*i*_ = 0.43 μM against SPU; **13**, *K*_*i*_ = 0.20 μM against PMU). The molecular modeling studies of compound **13** binding indicated that amino groups present in the inhibitor structure form hydrogen bonds with carbonyls of Ala170 and Ala366, while the phosphinic moiety interacts with both nickel(II) ions [27]. This type of binding in the bacterial urease active site makes the aminophosphinic class of inhibitors universal, contrary to structures designed to target the thiol group of cysteines, which in some pathogenic ureases are not present at locations that are relevant to enzyme activity [40, 41].

In the present research, the susceptibility of purified *H*. *pylori* urease to aminophosphonic inhibitors was studied. For this purpose, a highly efficient protocol was developed for *H*. *pylori* urease overexpression and extraction of the enzyme from *E*. *coli*. Again, as in other ureases, *N*-*n*-hexylaminomethyl-*P*-aminomethylphosphinic acid (**13**) and *N*-methylaminomethyl-*P*-hydroxymethylphosphinic acid (**4**) were found to be the most potent inhibitors, with *K*_*i*_ values of 0.294 μM and 1.032 μM, respectively. Acetohydroxamate efficiency was weaker (*K*_*i*_ = 23.2 μM). Recently, we showed that the two compounds **4** and **13** exceeded the ability of acetohydroxamic acid to prevent struvite formation in an artificial *Proteus mirabilis* urine infection model [42]. Therefore, whole-cell *H*. *pylori* urease inhibition studies were undertaken in the current project. Using an *E*. *coli* host, we have shown that the aminophosphinic class of inhibitors can cross bacterial cell membranes and, similarly to the results in the cell-free inhibition studies, inhibit urease activity with *IC*_50_ values in the micromolar range. Bis(aminomethyl)phosphinic acids exhibited the highest activity and were found to be more potent than the standard inhibitor, acetohydroxamic acid, regardless of the applied test conditions. In the whole-cell *H*. *pylori* J99 studies, the extended derivative **13** exerted an effect that was similar to those of most other compounds in the group, while the symmetrically substituted derivative **21** was the most efficient.

Structurally diverse inhibitors exhibited no or very low antiproliferative effects on normal human and animal cell lines. Taking into account the abovementioned properties of aminophosphinic acids, it could be concluded that this class of compounds constitutes a highly promising group of candidates for *H*. *pylori* treatment.

## Supporting information

S1 File Recombinant *Helicobacter pylori* urease purification. Detailed methodology and results.
(PDF)

S1 Table Inhibitory activity of the studied compounds against *Helicobacter pylori* urease. Lineweaver–Burk plots and *K*_*i*_ values.
(PDF)
