# Supplementary material for: Aminophosphinates against Helicobacter pylori ureolysis—Biochemical and whole-cell inhibition characteristics
Source: PLoS One. 2017 Aug 9;12(8):e0182437. doi: 10.1371/journal.pone.0182437 (PMC5550016; doi:10.1371/journal.pone.0182437)
Supplement: S1 File — Detailed methodology and results. (PDF) [file pone.0182437.s001.pdf]

S1. Recombinant *H. pylori* urease expression and purification.

The starter culture of recombinant *E. coli* strain Rosetta (DE3) + *pGEM::ureOP* was grown from cells stored in glycerol and inoculated at 1:1000 into 15 mL of LB broth supplemented with 100 μg/mL ampicillin. The culture was incubated at 37 °C for 16 h on a rotary shaker at 180 rpm. A 2% volume of the saturated pre-inoculum was introduced in 150 mL of LB medium (100 μg/mL ampicillin) in 250 mL Erlenmeyer flasks (OD_{600} ~0.1). Expression cultures were grown at 37 °C with intensive aeration (180 rpm) in LB medium and induced with IPTG (0.75 mM and Ni^{2+} 0.75 mM) in the exponential growth phase (usually at OD_{600} = 0.6). Further expression was conducted for 24 hours at 22°C. After harvesting by centrifugation (16 500 x g, 6 min with cooling), the cells were washed twice with 100 mL of 50 mM phosphate buffer (pH 7.0) supplemented with 50 mM Na_2SO_3, 1 mM EDTA, 1 mM β-mercaptoethanol, and 0.5 μM Ni^{2+} and subsequently disrupted ultrasonically in the same buffer. Soluble fractions were separated via double centrifugation (16 500 x g, 60 min, 4 °C). The supernatant (approximately 150 mL) was filtered through 0.45-μm PVDF filters and subjected to triple dialysis (dialyzed twice for 3 hours and finally overnight - in order to completely remove the ammonium salts) against phosphate buffer (pH 7.0) supplemented with 50 mM Na_2SO_3 and 1 mM EDTA (buffer A) using a membrane with a 10000-Dalton molecular weight cut-off (Spectra/Por Dialysis Membrane MWCO 10000). After passing through 0.45-μm PVDF filters, the crude extract was loaded onto a Q Sepharose XK 50/20 GE Healthcare anionic exchange column equilibrated with buffer A and eluted with a step gradient of NaCl (150 mM, 250 mM, and 450 mM NaCl in buffer A). The active fractions, eluted at a concentration of 250 mM NaCl, were pooled, and the ionic strength was raised to 1 M KCl. Pooled fractions containing urease were added to a Phenyl Sepharose XK 26/20 GE Healthcare

hydrophobic interaction column equilibrated with buffer A containing 1 M KCl and eluted with a decreasing linear gradient of KCl from 1 M to 0 M. Urease fractions, eluted with 200 mM KCl, were concentrated using an Amicon ultra filtration cell using a membrane with a 10000-Dalton molecular weight cut-off and then loaded onto a Sephacryl S300 XK 26/60 GE Healthcare gel filtration column equilibrated with buffer A containing 150 mM NaCl. The enzymatic activity fractions were subjected to triple dialysis (60 min, 4 °C, 2 500 x g) against buffer A (50 mM Na_2SO_3,1 mM EDTA, 50 mM phosphate buffer pH 7.0) using an Amicon ultrafiltration cell with membrane of 10000-Dalton molecular weight cut-off. After dialysis, fractions containing urease were added to a Mono Q 10/10 FPLC GE Healthcare anionic exchange column, equilibrated with buffer A, and developed using buffer A with an increasing linear gradient of NaCl from 0 to 1 M. Fractions containing urease were eluted with 200 mM NaCl and further purified to near homogeneity using a Superose 12 HR 10/30 FPLC GE Healthcare gel filtration column equilibrated with buffer A supplemented with 150 mM NaCl. Purified urease was dialyzed (twice for 3 hours and finally overnight) against a HEPES buffer (pH 7.5). The purification yield was 0.014 mg of purified urease per litre of growth medium. Protein concentration during preparation was measured using the Bradford method with bovine serum albumin as the standard [1]. Enzymatic activity of the purified urease was quantified spectrophotometrically using indophenol color reaction [2].

Table S1.1. Purification of recombinant *H. pylori* urease. Procedure was based on five different chromatographic steps.

Purification step	Protein [mg]	Total activity [Units]	Specific activity [Units/mg]	Purification fold [-]
Crude extract	691.7	45098	65.2	1
Q-Sepharose column	83.2	27132	326	5.0
Phenyl Sepharose column	20.0	13446	672	10.3
Sephacryl S-300 column	10.4	11731	1128	17.3
Mono Q column	1.6	2175	1360	20.9
Superose 12 column	0.86	1422	1654	25.4

In this study, several chromatographic beds were analyzed for their suitability for the purification of *H. pylori* urease (Table S1.1). During each step, recombinant *H. pylori* urease eluted with slightly different conditions than those observed for the native enzyme from *Sporosarcina pasteurii* and *Proteus mirabilis* [3, 4], mainly at different ionic strengths of the elution buffers. Similar to the native urease from *H. pylori*, the recombinant enzyme comprised two distinct subunits of 61.7 and 29.5 kDa (Fig. 3). The applied multi-step chromatography yielded *H. pylori* urease with high a homogeneity and a specific activity of 1654 U/mg (25.4% recovery).

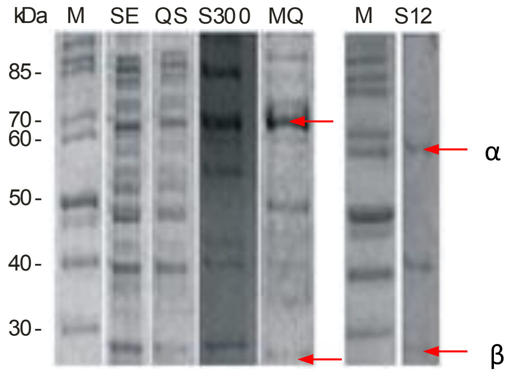

Figure S1.1. SDS-PAGE electrophorograms of recombinant *H. pylori* urease fractions obtained during the multistep purification procedure. The red arrows indicate the α and β subunits of urease with molecular weights of 61.7 and 29.5 kDa, respectively. M – molecular weight marker, SE – a soluble fraction of proteins before purification, QS – enzymatically active fractions after performing the ion-exchange step in the Q-Sepharose column, S-300 - urease fractions after size exclusion chromatography, Sephacryl S-300 column, MQ – urease fractions obtained in additional ion-exchange chromatography steps using a Mono Q column, S-12 – final urease preparation obtained in the last step of gel filtration using a Superose S-12 chromatographic medium.

The purified fractions of enzyme were subjected to standard biochemical analysis in order to establish pH-stability of urease and its affinity to urea. Urease activity was measured by means of indophenol method [2] in 5 minutes assays at 37 °C. Studies on the pH dependence of urease activity were performed in following buffers: 3 mM phosphate (pH 5.0-8.3), 3 mM AMPSO (pH 8.0-9.0), and 3 mM bicarbonate buffer (pH 9.0-10.0) (Figure S1.2 panel A). The kinetic parameters of urease in noninhibited reaction, the Michaelis constant K_M and maximum reaction rate V_{max}, were determined by measuring initial rates of the reactions (Figure S1.2 panel B). The kinetic characteristics of the *H. pylori* urease were as follows: K_M of 0.28 ± 0.017 mM, V_{max} of 1.312 ± 0.074 µM s^{-1} and k_{cat} of $2.9 \cdot 10^3$ s^{-1}. The pH optimum of the enzymatic reaction was observed in the range of 7.4–7.8.

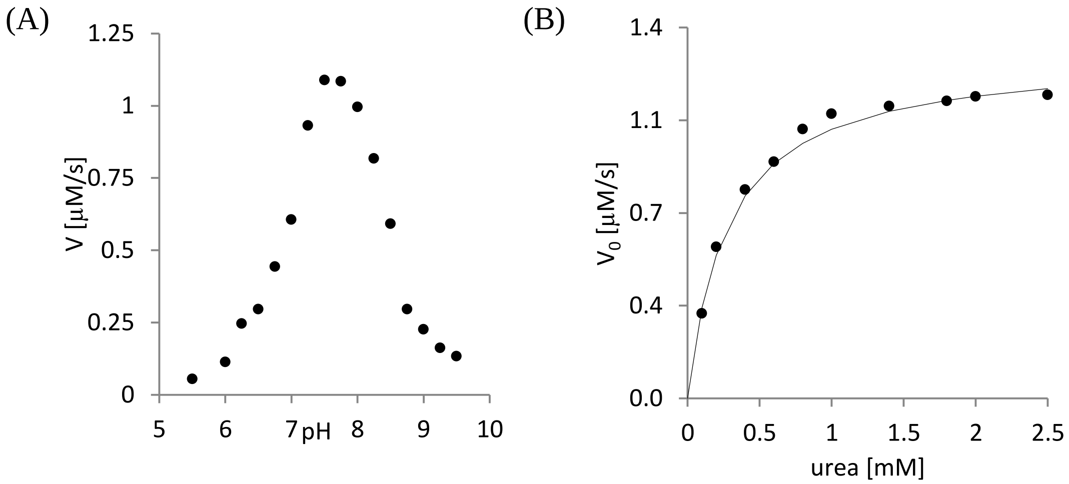

Figure S1.2. Analysis of catalytic activity of a highly purified preparation of *H. pylori* urease. Determination of the optimum pH (A) as well as K_m and V_{max} of enzymatic reaction (B).

Recombinant Helicobacter pylori urease purification with the use of affinity chromatography. The results reported by Icatlo et al. [6] offered a simple method for native urease affinity purification directly from crude extracts using heparinoid (Cellufine sulfate) gel. The method was based on a two-step procedure using the same column bed that takes advantage of the differential affinity of urease to heparinoid at two hydrogen ion concentrations. This procedure used for the preparation of the recombinant enzyme was found ineffective, as very little contaminating protein was removed. Affinity chromatography was performed using Cellufine Sulfate (Chisso, Japan). The plasmid encoded urease expression was conducted as above. After passing through 0.45 µm PVDF filters, the crude cell extract (about 10 mL) was loaded at a flow rate of 3 mL/min onto the step A gel column (14-mm diameter, 15 cm of Cellufine sulfate bed height) equilibrated with 20 mM phosphate buffer, pH 6.5 (PB65). Elution was done with PB65 (10 x column volumes). Fractions containing urease eluted as the first peak (unbound fraction), whereas more strongly adhered contaminants were washed out with elution buffer. Urease containing fractions were then pooled together, adjusted to pH 5.5 and loaded at a flow rate of 3 mL/min onto the step B column

(14-mm diameter, 15 cm of Cellufine sulfate bed height) pre-equilibrated with 20 mM phosphate buffer, pH 5.5 (PB55). The column was washed with the same phosphate buffer (10 x column volumes). Elution of protein was done with 20 mM phosphate buffer pH 7.4 using linear gradient of NaCl (0.15M – 0.8M). Urease fractions, eluted with 0.15 M NaCl, were pooled, concentrated and dialyzed (twice for 3 hours and finally overnight) against 50 mM HEPES buffer pH 7.5.

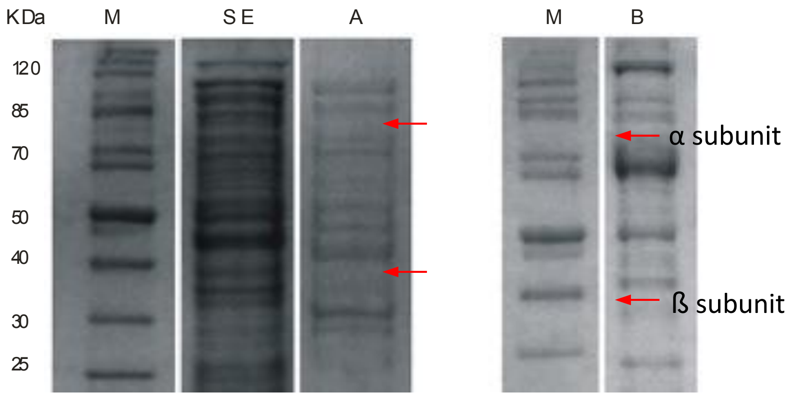

Figure S1.3. SDS-PAGE electrophorograms of partially purified *Helicobacter pylori* urease fractions obtained during affinity purification with the use of Cellufine sulfate. The red arrows indicate α, β subunits of urease with molecular weight of 61.7 and 29.5 kDa, respectively. M- molecular weight marker, SE – proteins before purification, A – enzymatically active urease eluted from step A column at pH 6.5, B - eluted from step B column.

References

1. Bradford MM. A rapid and sensitive for the quantitation of microgram quantitites of protein utilizing the principle of protein-dye binding. Anal Biochem 1976;72:248-254.
2. Weatherburn MW. Phenol-Hypochlorite Reaction for Determination of Ammonia. Anal Chem 1967;39:971-974.
3. Berlicki L, Bochno M, Grabowiecka A, Białas A, Kosikowska P, Kafarski P. N-substituted aminomethanephosphonic and aminomethane-P-methylphosphinic acids as inhibitors of ureases Amino Acids 2012;42:1937-45.
4. Breitenbach JM, Hausinger RP. *Proteus mirabilis* urease partial purification and inhibition by boric acid and boronic acids. Biochem J 1988;250:917-920.
5. Icatlo FCJr, Kuroki M, Kobayashi C, Yokoyama H, Ikemori Y, Hashi T, Kodama Y. Affinity purification of *Helicobacter pylori* urease J Biol Chem 1998;273:18130-18138.
