# Supplementary material for: Aminophosphinates against Helicobacter pylori ureolysis—Biochemical and whole-cell inhibition characteristics
Source: PLoS One. 2017 Aug 9;12(8):e0182437. doi: 10.1371/journal.pone.0182437 (PMC5550016; doi:10.1371/journal.pone.0182437)

Table S1. Inhibitory activity (K_i values) of the studied phosphinic and phosphonic acid based compounds against purified recombinant *Helicobacter pylori* urease.

<table>
<tr><th>K_i values of compounds</th><th>Lineweaver-Burk plot for inhibition of H. pylori urease by studied phosphinic and phosphonic acid based compounds</th></tr>
<tr><td>Inhibitor 1
$K_i = 38.3 \pm 1.1$ μM</td><td>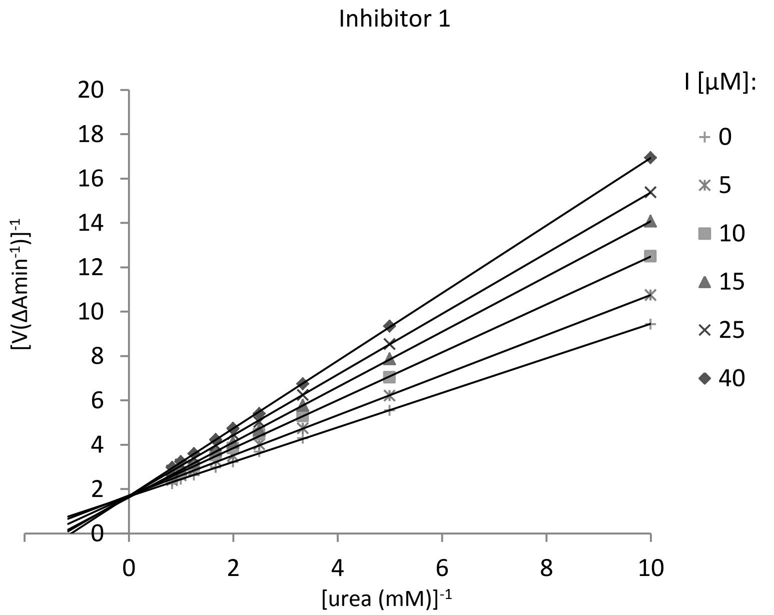
</td></tr>
<tr><td>Inhibitor 2
$K_i = 61.6 \pm 3.5$ μM</td><td>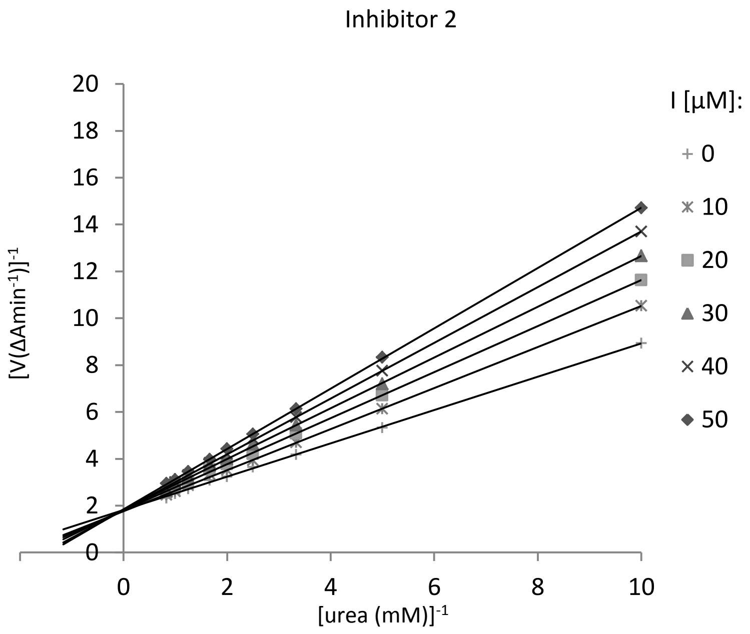
</td></tr>
</table>

Inhibitor 3

$K_i = 9.27 \pm 0.35$ μM

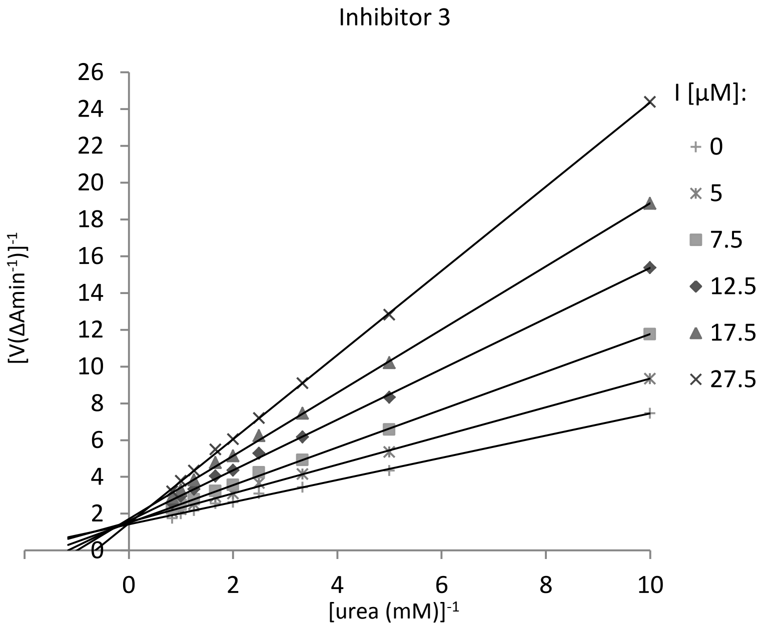

Inhibitor 4

$K_i = 1.032 \pm 0.068$ μM

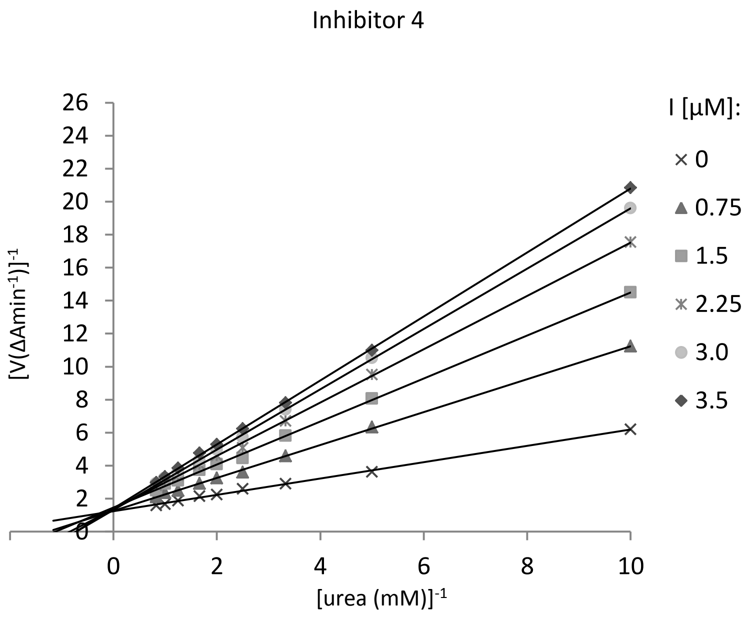

Inhibitor 5

$K_i = 74.3 \pm 4.7$ μM

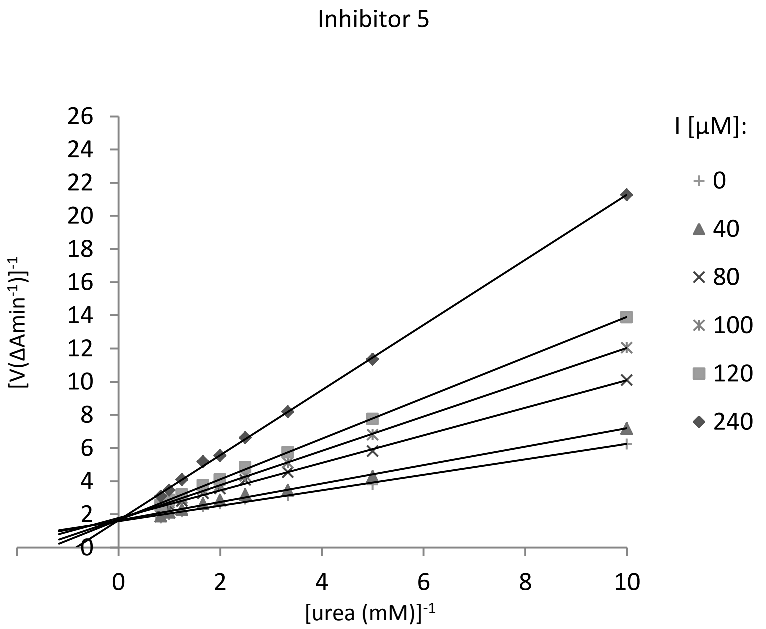

Inhibitor 6

$K_i = 61.0 \pm 9.2$ μM

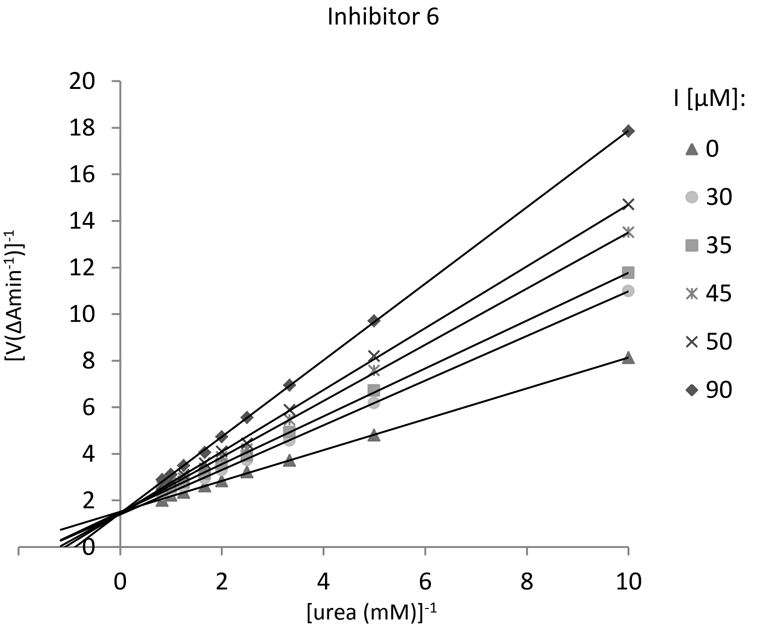

Inhibitor 7

$K_i = 20.9 \pm 2.0\ \mu M$

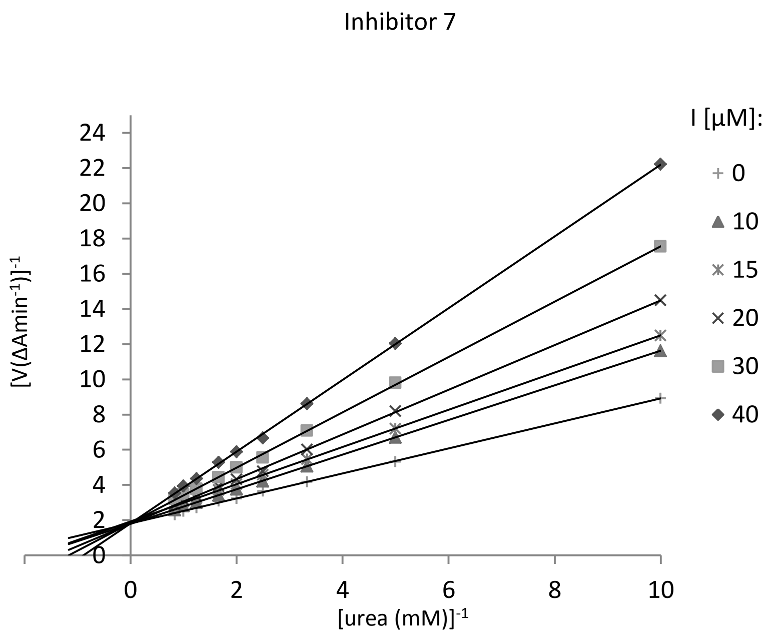

Inhibitor 8

$K_i = 29.9 \pm 2.0\ \mu M$

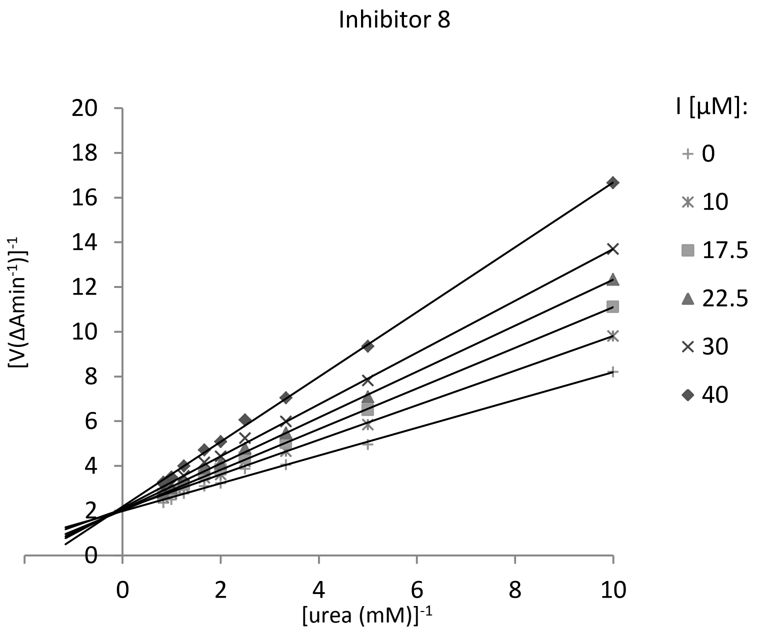

Inhibitor 9

$K_i = 22.1 \pm 2.1\ \mu M$

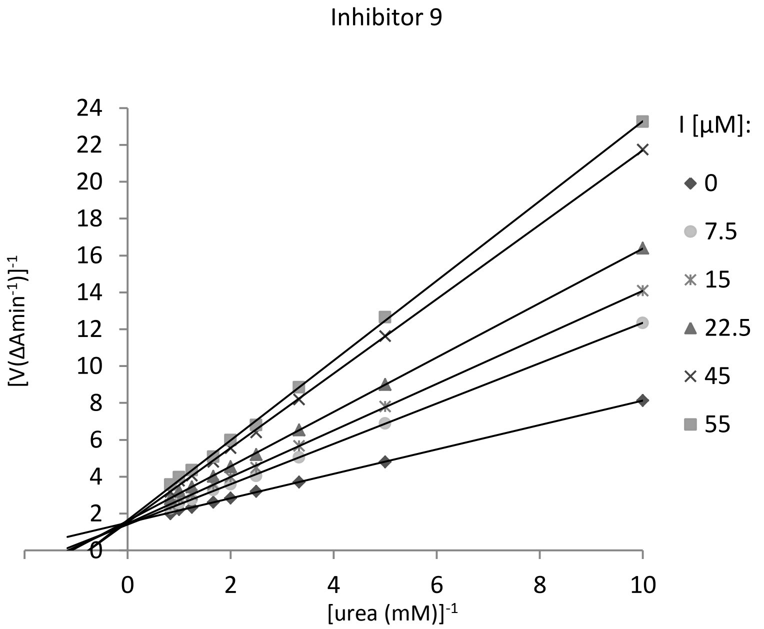

Inhibitor 10

$K_i = 440 \pm 70$ µM

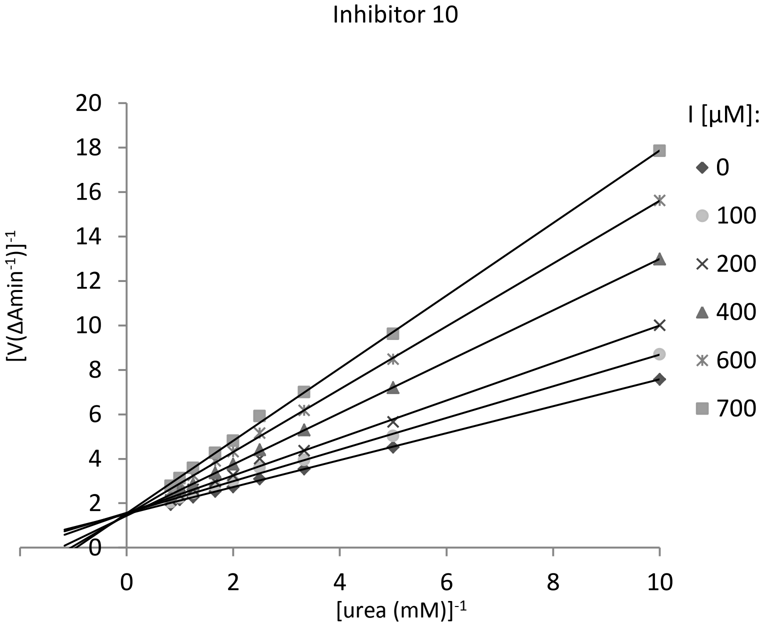

Inhibitor 11

$K_i = 27.0 \pm 2.1$ µM

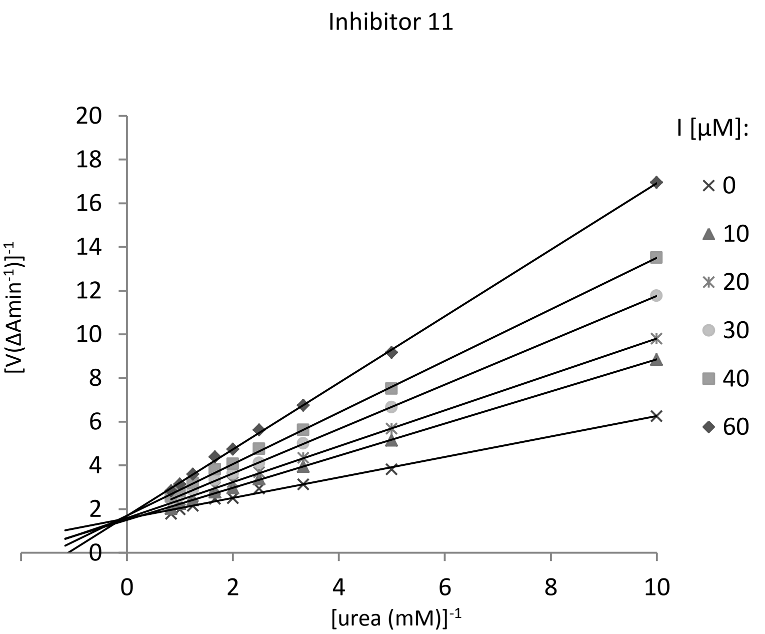

Inhibitor 12

$K_i = 43.1 \pm 3.6\ \mu M$

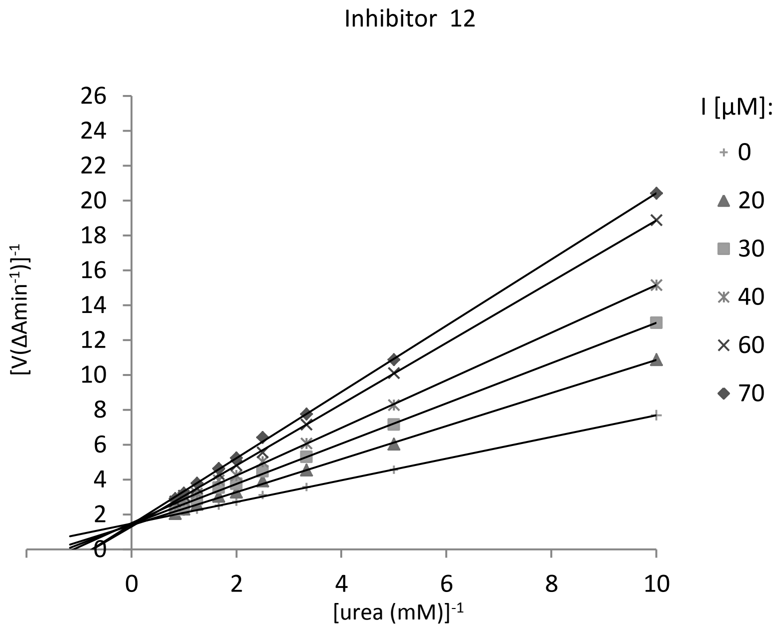

Inhibitor 13

$K_i = 0.294 \pm 0.013$ μM

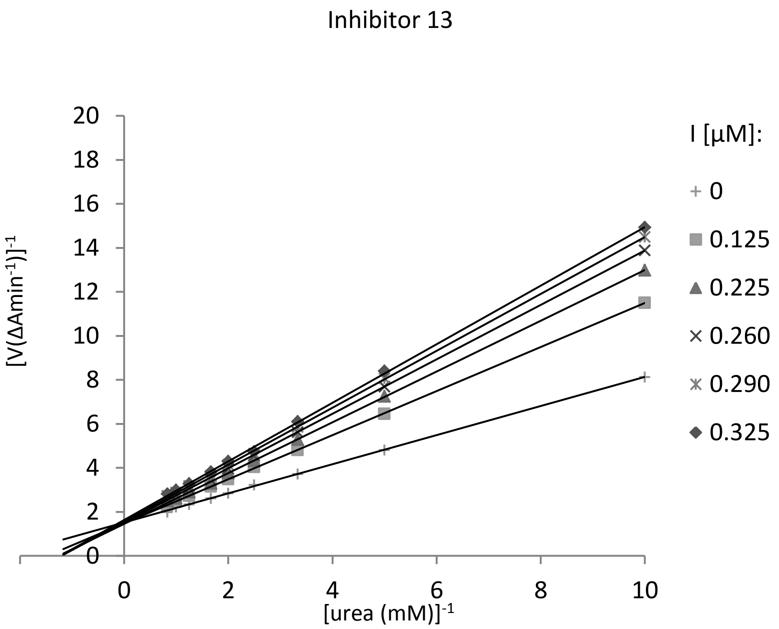

Inhibitor 14

$K_i = 878 \pm 25$ μM

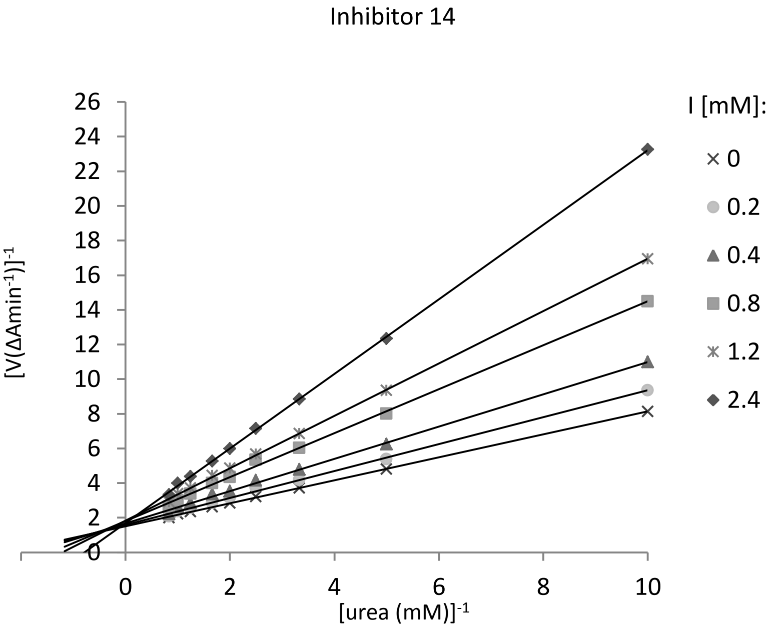

Inhibitor 18

$K_i = 44.4 \pm 2.5$ μM

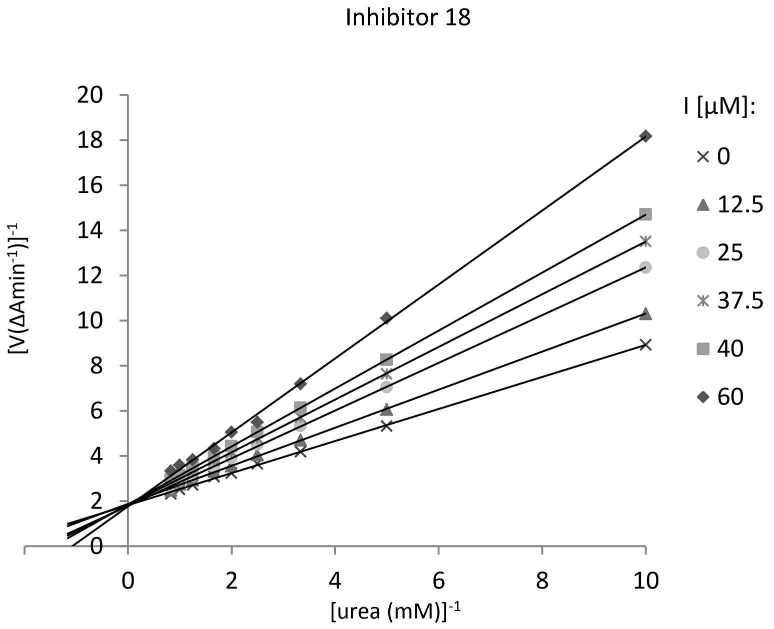

Inhibitor 19

$K_i = 36.4 \pm 4.9$ μM

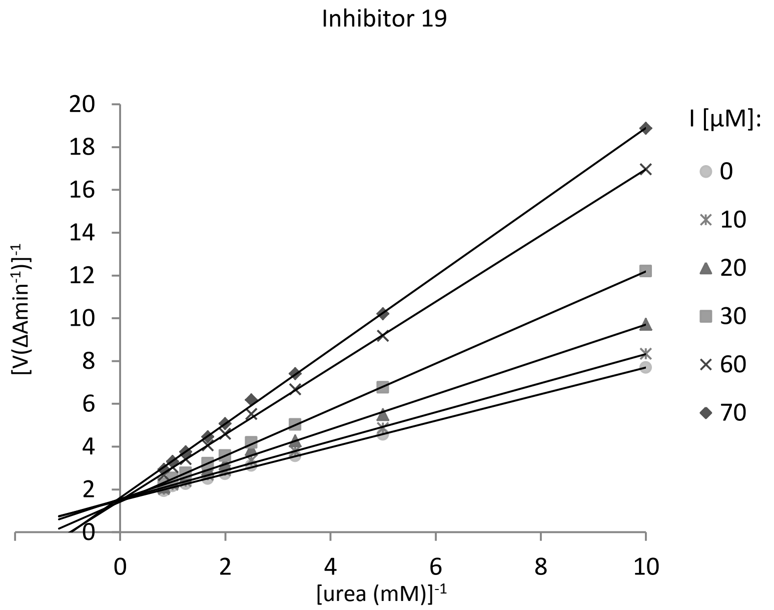

Inhibitor 21

$K_i = 26.1 \pm 1.8\ \mu M$

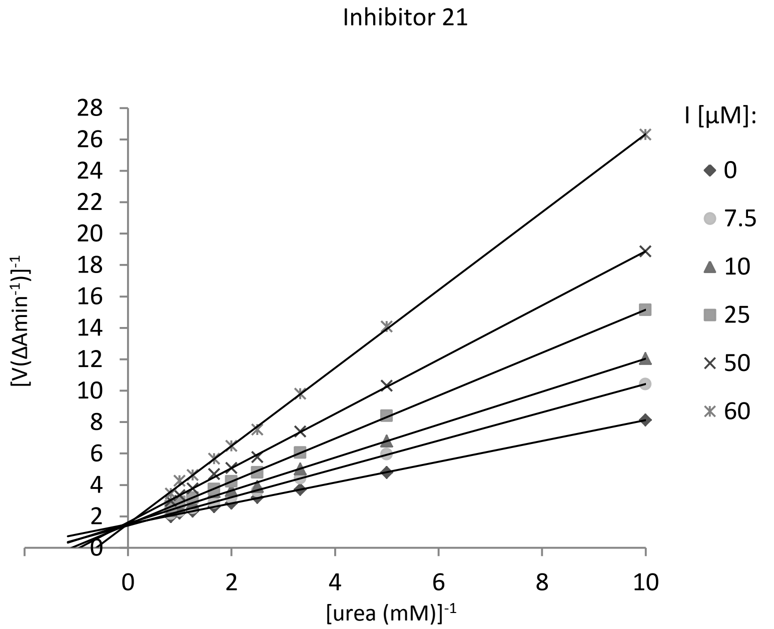

Inhibitor 23

$K_i = 50.7 \pm 4.8\ \mu M$

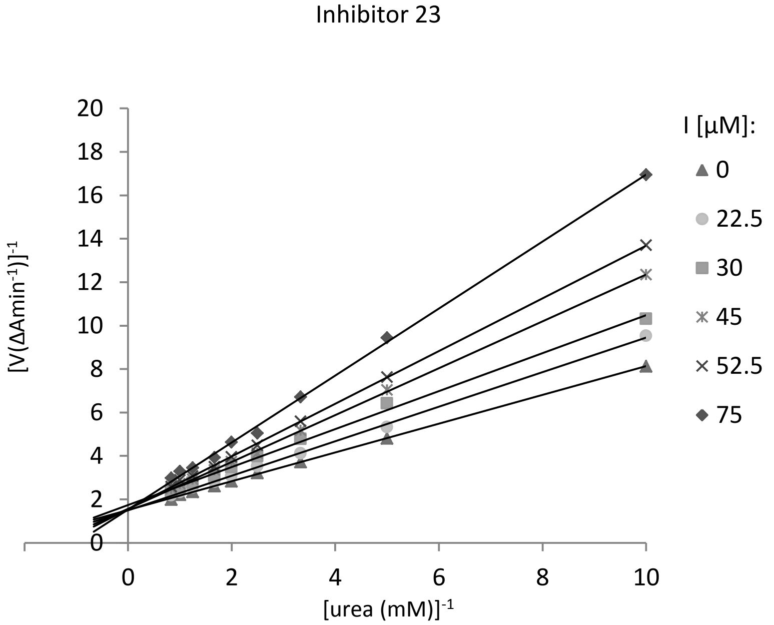

Inhibitor 24

$K_i = 23.2 \pm 1.5$ μM

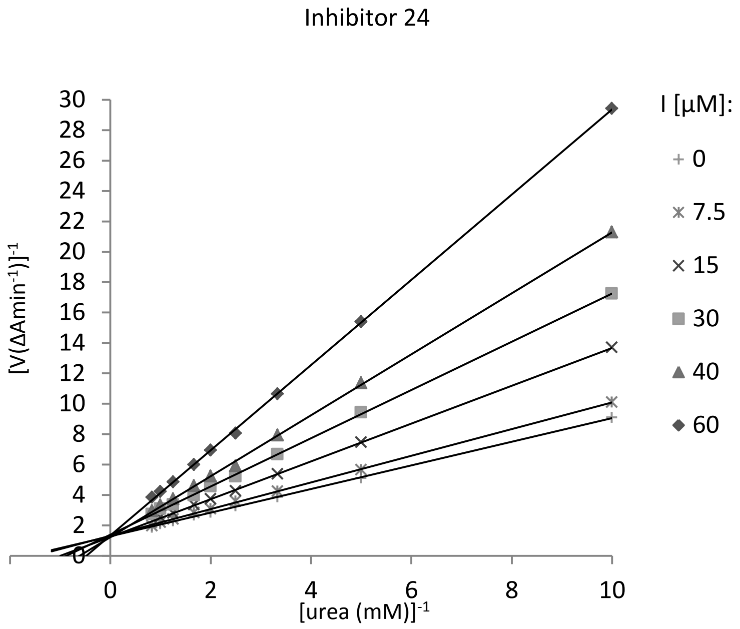

Supplement: S1 Table — Lineweaver–Burk plots and Ki values. (PDF) [file pone.0182437.s002.pdf]
